# Recent progress in epigenetics of obesity

**DOI:** 10.1186/s13098-022-00947-1

**Published:** 2022-11-17

**Authors:** Feng-Yao Wu, Rui-Xing Yin

**Affiliations:** 1 Department of Comprehensive Internal Medicine, Affiliated Infectious Disease Hospital of Nanning (The Fourth People’s Hospital of Nanning), Guangxi Medical University, No. 1 Erli, Changgang Road, Nanning, 530023 Guangxi People’s Republic of China; 2 Department of Cardiology, Institute of Cardiovascular Diseases, The First Affiliated Hospital, Guangxi Medical University, 6 Shuangyong Road, Nanning, 530021 Guangxi People’s Republic of China

**Keywords:** Obesity, Epigenetics, DNA methylation, Histone modification, miRNA

## Abstract

Nowadays, obesity is one of the largest public health problems worldwide. In the last few decades, there has been a marked increase in the obesity epidemic and its related comorbidities. Worldwide, more than 2.2 billion people (33%) are affected by overweight or obesity (712 million, 10%) and its associated metabolic complications. Although a high heritability of obesity has been estimated, the genetic variants conducted from genetic association studies only partially explain the variation of body mass index. This has led to a growing interest in understanding the potential role of epigenetics as a key regulator of gene-environment interactions on the development of obesity and its associated complications. Rapid advances in epigenetic research methods and reduced costs of epigenome-wide association studies have led to a great expansion of population-based studies. The field of epigenetics and metabolic diseases such as obesity has advanced rapidly in a short period of time. The main epigenetic mechanisms include DNA methylation, histone modifications, microRNA (miRNA)-mediated regulation and so on. DNA methylation is the most investigated epigenetic mechanism. Preliminary evidence from animal and human studies supports the effect of epigenetics on obesity. Studies of epigenome-wide association studies and genome-wide histone modifications from different biological specimens such as blood samples (newborn, children, adolescent, youth, woman, man, twin, race, and meta-analysis), adipose tissues, skeletal muscle cells, placenta, and saliva have reported the differential expression status of multiple genes before and after obesity interventions and have identified multiple candidate genes and biological markers. These findings may improve the understanding of the complex etiology of obesity and its related comorbidities, and help to predict an individual’s risk of obesity at a young age and open possibilities for introducing targeted prevention and treatment strategies.

## Introduction

Obesity is an abnormal or excessive accumulation of fat mass. It may also lead to the development of many related comorbidities, such as blood lipid disorder, high blood pressure, insulin resistance, type 2 diabetes (T2D), metabolic syndrome (MetS), cardiovascular disease, hepatic steatosis, and others [1]. The prevalence of obesity is reaching unprecedented levels and will continue to increase in modern society [2]. It is estimated that 58% of adults worldwide will reach the diagnostic criteria for obesity by 2030 [3], and obesity has become an epidemic with the fourth highest risk factor associated with disability-adjusted life years [4]. Obesity is a complex genetic disease caused by the interaction of genetic predisposition, epigenetics, metagenomics, and environmental risk factors [5].

**Fig. 1:**
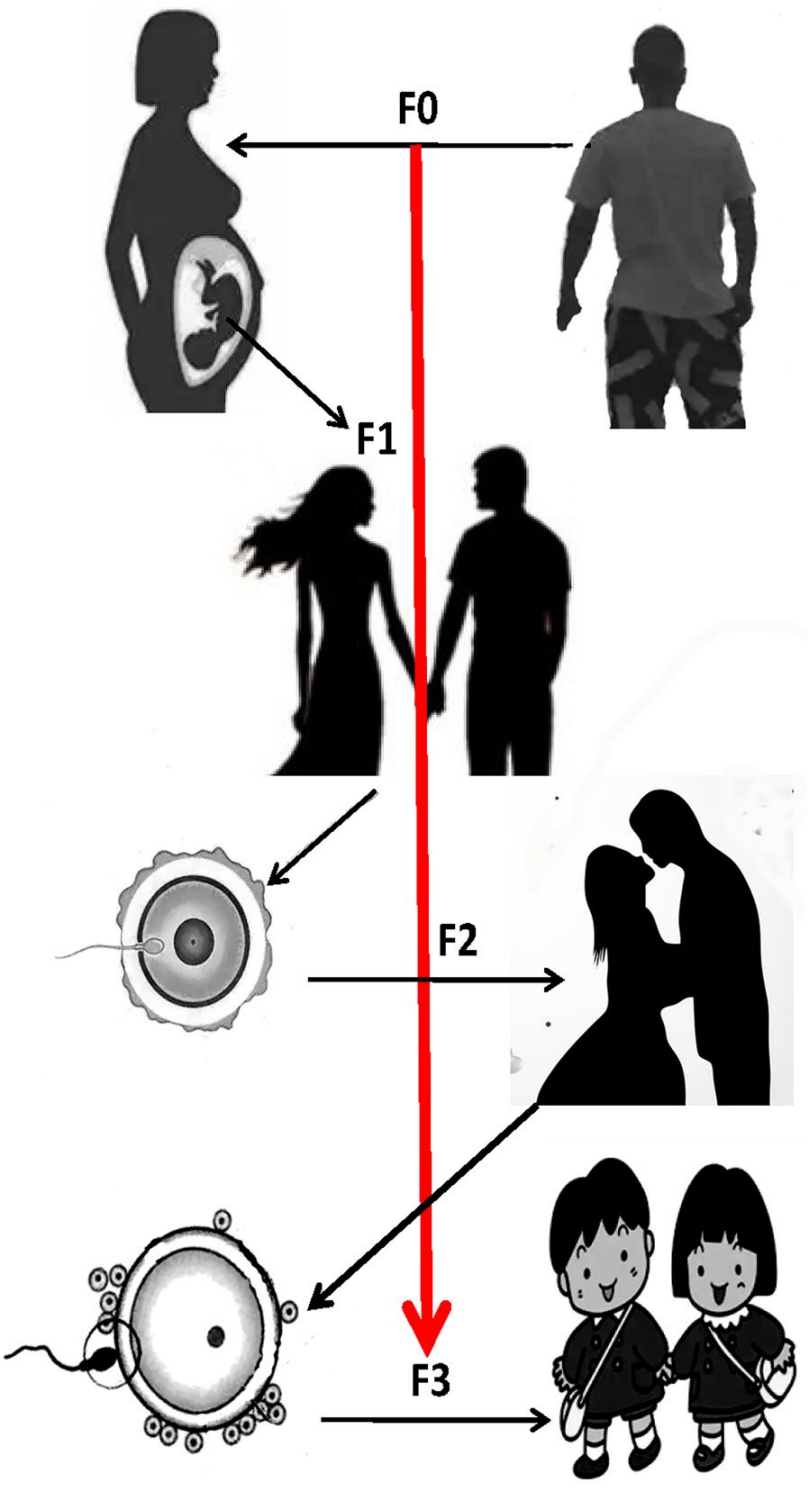
Transgenerational transmission of inherited epigenetic changes. It generally involves exposure of F1 gametes in utero to maternal experience (F0), which subsequently affects F2 offspring, and then transmission of the epigenetic characteristics of F3 offspring via unexposed F2 gametes

**Fig. 2:**
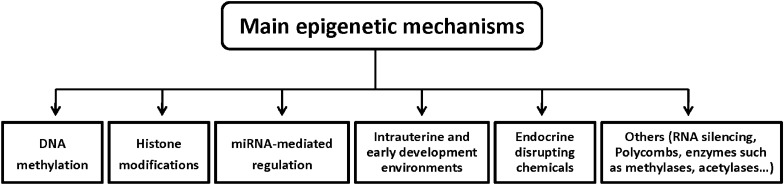
The main mechanisms involved in epigenetic regulation

Epigenetics is a subdiscipline of genetics that studies heritable changes in gene expression in the absence of changes in the nucleotide sequence of genes [5–7]. Epigenetics is a discipline that links environmental factors to patterns of genetic change, such as between rapid changes in dietary habits and the observed obesity phenotype. DNA methylation (DNAm), as a key part of epigenetics, may be the mechanism linking obesity and clinical manifestations. Research related to epigenetics or its role on metabolic disease is still an emerging area of research, but it is shining brightly, attracting a lot of attention and growing rapidly. We can think of epigenetic modifications as differential packaging of DNA under different conditions, and this approach can control (allow or silence) the expression of certain genes in specific tissues [8]. Lifestyle, dietary pattern, gut microbiota, and other environmental factors, can affect epigenetic programming through the various periods of life, especially parental gametes, fetus and early postnatal development [9]. Epigenetics is usually classified according to the degree of dependence of genes on genetic changes. There are 3 classes of epigenetic variation [10]. (1) Obligate epigenetic variants reflect complete dependence on genetic variation and there is a strict one-to-one correspondence between the epigenotype and either *cis*- or *trans*-acting genetic variation (eg, transposon insertions). The epigenotype of the locus is strictly determined by genotype, and it is also an obligatory phenotype of the alternative genotypes; (2) facilitative epigenetic variants reflect semi-independence on changes in genetic material (eg, retrotransposon insertions), the genotype directs or potentiates the epigenotype in a probabilistic but not strictly deterministic manner; and (3) pure epigenetic variants have no genetic changes, it is generated by stochastic events that are largely independent of genetic variation, stochastic events generate alternative epialleles at some finite frequency regardless of the genotype [10]. Epigenetic states can be transferred through meiosis and mitosis (sperm cells and oocytes). Mitosis can maintain epigenetic changes throughout the cell cycle, and meiosis can transfer epigenetic changes across generations [11]. Epigenetic changes that occur when the epigenome is reinfluenced by an environmental event are considered to be acquired. The effects of acquired epigenetic changes can be intragenerational or intergenerational. Where intragenerational epigenetic change occurs during the life cycle of the affected individual and leads to later life changes; however, intergenerational epigenetic change occurs only with in the gametes of the fetal life stages and is not transmitted to posterity [12]. Transgenerational transmission of inherited epigenetic changes generally involves exposure of F1 gametes in utero to maternal experience (F0), which subsequently affects F2 offspring, and then transmission of the epigenetic characteristics of F3 offspring via unexposed F2 gametes (Fig. 1) [12]. So far, the main epigenetic mechanisms known include DNAm, histone modifications, and microRNA (miRNA)-mediated regulation and so on (Fig. 2). These genetic alterations can be mediated through transfer of mitosis (via cytokinesis) or meiosis (transgenerational inheritance) [8, 11]. Nowadays, after the analysis of the human genome sequence has been completed, finely regulated chromatin epigenetic networks, DNAm and histone modifications needed to be studied to clarify how the same DNA sequences give rise to different cells, lineages, and organs, i.e., the different phenotypes [13]. The first epigenetic study in humans was small in the scale and studied only a limited number of gene loci. Recently, with advances in high-throughput technologies and increased affordability, humans have performed massive epigenome-wide association studies (EWAS) and integrated genomic information at different layers. This has allowed us to study the interactions between genotypes, epigenomes, transcriptomes, and environmental factors [14–16]. DNAm is the best-studied epigenetic modification. It is also a key and stable epigenetic mechanism and locus-specific DNAm levels associated with obesity and cardiometabolic traits through genome-wide, genetic variation, and candidate gene approaches [7, 17].

Although several review articles on epigenetics of obesity have been published in recent years [5, 8, 9, 17–19], these published papers often include only one or two mechanisms of epigenetics of obesity. There are still few systematic and comprehensive review papers in this field. Therefore, this article aims to review the current literature on epigenetic changes in obesity. It includes DNA methylation in different biological specimens such as blood samples (newborn, children, adolescent, youth, woman, man, twin, race, and meta-analysis), adipose tissues, skeletal muscle cells, placenta, and saliva; histone modifications; miRNA-mediated regulation; the influence of intrauterine environment and early development environment; as well as the effects of endocrine disrupting chemicals. The identification of obesity-related DNAm changes in different populations may help link obesity with its associated clinical symptoms early in the disease, explore potential mechanisms, and may provide new targets for early prevention of obesity.

## DNAm

**Fig. 3:**
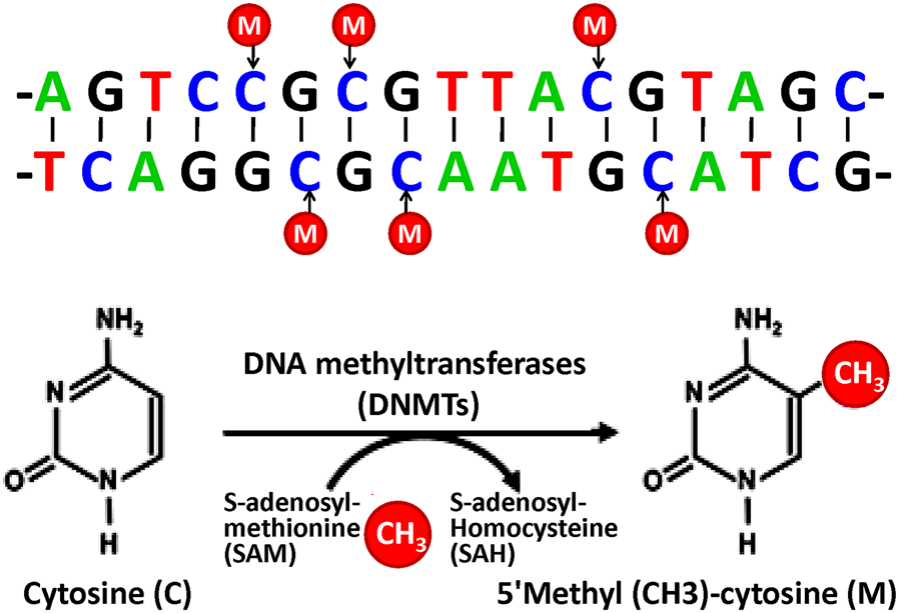
Diagram showing the mechanism of DNA methylation (DNAm). DNAm often takes place at the 5-carbon location of the cytosine (C) base, mainly in the CG position (CpG loci), which are unevenly allocated throughout the genome and to a lesser extent in non-CG context. Several DNA methyltransferases (DNMTs) are responsible for ligating methyl groups to DNA. The methyl donor is S-adenosylmethionine (SAM). SAM is converted to S-adenosylhomocysteine (SAH)

**Fig. 4:**
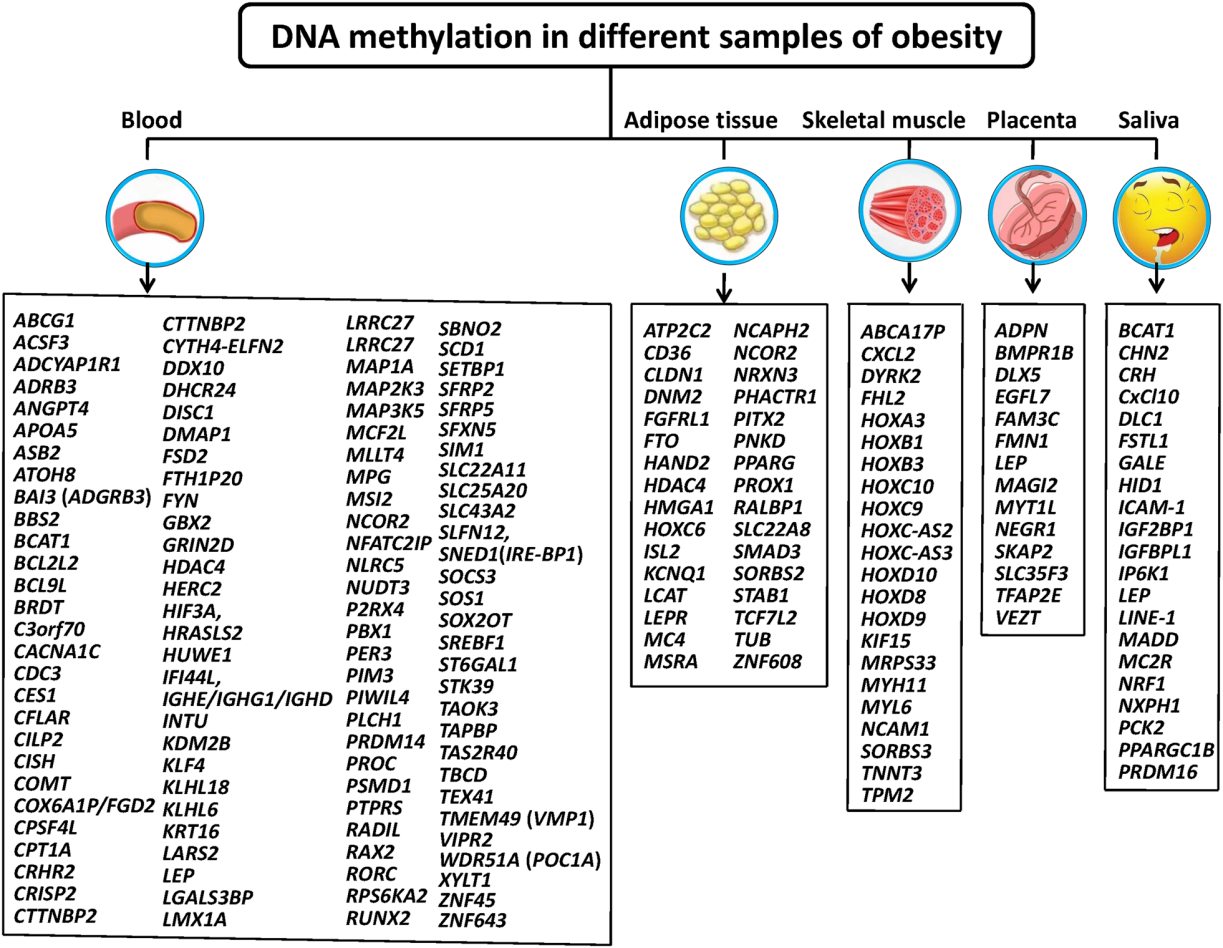
DNA methylation (DNAm) in different biological specimens of obesity. The figure illustrates tissues and genes with observed alterations in DNAm in subjects with obesity. Some of these genes are also associated with differential gene expression. Changes in DNAm are more often in obese individuals than in non-obese participants. The full name of the abbreviations can be found in the abbreviate table

DNAm, a dynamic process that controls genome integrity and transcriptional activity is the most studied epigenetic mechanism. DNAm takes place at the 5-carbon location of the cytosine (C) base, mainly in the CG position (CpG loci), which are unevenly allocated throughout the genome and to a lesser extent in non-CG context. Several DNA methyltransferases (DNMTs) are responsible for ligating methyl groups to DNA. These DNMTs include DNMT 3 beta (DNMT3B), DNMT 3 alpha (DNMT3A), and DNMT1 (Fig. 3). These CpG loci are often found in the promoter regions of genes, and the addition of methyl becomes a steric barrier to transcription factor ligation and gene expression: hypermethylation is often related to transcriptional repression, whereas hypomethylation is related to the activation [6, 8]. The methyl donor is S-adenosylmethionine. There are active demethylation (by translocation enzymes, possibly oxidizing methyl groups to hydroxymethylation, and then repair) and passive demethylation. Furthermore, when methylated cytosine is first oxidized to hydroxymethyl-cytosine by translocation enzymes, targeted passive demethylation also occurs. This state remains until the next S-phase. During this period, hydroxymethyl-cytosine is not identified by DNMT1 and thus changes into an unmethylated cytosine on the newly synthesized strand. Thus, translocases can target cis-regulatory elements to lose methylation at specific loci [19]. Methylation changes in candidate genes are associated with growth, circadian clock regulation, immunity, inflammation, appetite control, metabolism, insulin signaling, and obesity or related phenotypes [8, 9]. Currently, the most commonly used biological samples for DNAm detection are blood samples including whole blood and leukocytes [20–53], adipose tissues [16, 54–59], skeletal muscles [60–63], placenta [64–68] and saliva [69–73] etc. (Fig. 4).

### DNAm in blood samples

#### DNAm in newborns

Martin et al. [20] identified CpG loci in cord blood leukocytes using data on 173 mother-male and 187 mother-female from the Newborn Epigenetics Study (NEST) cohort. The results showed that maternal pre-pregnancy obesity was associated with 293 CpG loci in male offspring and 876 CpG loci in female offspring (false discovery rate, FDR < 5%). In female offspring, 57 CpG loci, including the top 18, were assigned to tapasin binding protein (*TAPBP*, methylation changes: -0.83% to 4.02%). CpG methylation differences for *TAPBP* were also found in male offspring (change: -0.30% to 2.59%), but none of the CpG loci were replicated in the Avon Longitudinal Study of Parents and Children (ALSPAC) cohort. In NEST, differences in the methylation of *TAPBP*’s CpG loci were related to body mass index z-scores (BMIz, cg23922433 and cg17621507) [20]. This suggests that maternal obesity may have gender-specific differences in the BMIz of offspring (Table 1). Jönsson et al. [21] also explored whether lifestyle interventions in obese pregnant women affect epigenetic variation in umbilical cord blood as well as body composition of offspring. They performed a genome-wide DNAm analysis of 208 offspring from the Treatment of Obesity Pregnant Women study. As compared to controls (standard of care), the offspring of mothers had alterations in DNAm at 379 loci in cord blood, annotated at 370 genes, after a lifestyle intervention such as physical activity with or without dietary recommendations. A total of 370 genes including response to fatty acids and development of adipose tissue were overrepresented in terms of Gene Ontology. The offspring of mothers who receive lifestyle interventions had more lean body mass at birth than the control group. DNAm was found at 17 loci, mainly annotated 4 genes, including the disrupted-in-Schizophrenia-1 (*DISC1*), gastrulation brain homeobox 2 (*GBX2*), HECT, UBA and wwe domains containing E3 Ubiquitin ligase 1 (*HUWE1*), and HECT and RLD domain containing E3 ubiquitin protein ligase 2 (*HERC2*), partially mediated the effect of lifestyle intervention on offspring lean body mass (FDR < 5%). Furthermore, 22 methylation loci were associated with offspring BMIz within 3 years after birth (*P* < 0.05) [21]. These findings suggest that lifestyle intervention is associated with epigenetic changes in offspring that can affect offspring lean body mass and early growth.

**Table 1:** Epigenome-wide DNA methylation association analyses of obesity in different populations

References	Sample size	Tissue	Phenotype	Gene	CpG number
Martin et al. [20]	187 mother-female /173 mother-male offsprings	Cord blood leukocytes	BMI z-score (BMIz)	*TAPBP* (female offspring)	876; 293
Jönsson et al. [21]	208	Cord blood	BMIz	*DISC1, GBX2, HERC2, HUWE1*	379
Huang et al. [22]	78	Whole blood	BMIz	*FYN, PIWIL4, TAOK3*	129 differentially methylated CpG
Fradin et al. [23]	40	Whole blood cells	BMI, BMIz	*LMX1A, ACSF3*	31 CpGs (obesity); 151 (severe obesity, > 10%)
Rzehak et al. [24]	374	Whole blood	BMI, fat-mass, fat-free-mass, fat-mass-index, fat-free-mass-index	*SNED1(IRE-BP1), KLHL6, WDR51A(POC1A), CYTH4-ELFN2, CFLAR, PRDM14, SOS1, ZNF643(ZFP69B), ST6GAL1, C3orf70, CILP2, MLLT4*	BMI (212), fat-mass (230), fat-free-mass (120), fat-mass-index (24), fat-free-mass-index (15)
Li et al. [25]	3 obese cases, 4 controls; 39 obese cases, 42 controls	Peripheral blood leukocytes	BMI	*HDAC4, RAX2; SLC25A20; APOA5* and *CES1* (negative correlation)	4 85 000 methylation sites, 226 methylated CpGs
Samblas et al. [26]	24; 91	White blood cells	BMIz	*VIPR2, GRIN2D, ADCYAP1R1, PER3, PTPRS; PTPRS, PER3* with BMI z-score	734
Huang et al. [27]	265	Leukocytes	BMIz	*ATOH8, CDC37/MIR1181, COMT/TXNRD2, DDX10, TAS2R40*, *TBCD*	6 (mid-childhood)
He et al. [28]	263	Peripheral leukocytes	BMI	*SIM1*	28
Sherwood et al. [29]	297	Peripheral blood	BMI trajectories	*LEP*	23; cg23381058 (early transient overweight trajectory), cg05091920 (early persistent obesity trajectory)
Wang et al. [30]	325	Whole blood	BMI status transition	*P2RX4, RADIL*	174
Vehmeijer et al. [31]	4133 children from 23 studies	Cord blood and whole blood	BMI	*SFRP5, SLC43A2, SFXN5*	187
Xu et al. [32]	48 obese, 48 lean	Peripheral blood samples	BMI	-	23, 305
Wang et al. [33]	700, 188, 2097	Neutrophils, Leukocytes	BMI	*HRASLS2, SOCS3*, *CISH*, *PIM3*, *KLF4, HRASLS2*	76 (novel 29)
Wilson et al. [34]	871 women, 187 women	Blood samples	BMI	*ANGPT4, RORC, SOCS3, FSD2, XYLT1, ABCG1, STK39, ASB2, CRHR2*	4 CpG sites FDR *q* < 0.05
Kvaløy et al. [35]	60 lean, 60 obese	Leucocyte	BMI	*TEX41, IGHE/IGHG1/IGHD, DMAP1, SETBP1* (novel findings); *COX6A1P/FGD2, SBNO2, RPS6KA2, SOCS3*	10 differentially methylated CpG sites
Shen et al. [36]	927 Mexican American women	Blood DNA samples	BMI, Weight change	*HIF3A*	3 (position 46,801,557, 46,801,642, 46,801,699)
Guay et al. [37]	61 30	Blood and visceral adipose tissue (VAT)	Waist-to-hip ratio	*ADRB3*	-
Skuladottir et al. [38]	16	Whole blood samples	Weight	*SCD1*	3 (cg00954566, cg24503796, cg14089512)
Keller et al. [39]	120 (90% men)	Blood samples	BMI, Weight-loss	*LRRC27, CRISP2, SLFN12, NUDT3, NCOR2*	15 (negatively correlated with weight change)
Ollikainen et al. [40]	30 twin pairs	Leukocyte DNA	BMI	-	No differentially methylated CpG sites between co-twins discordant for BMI
Li et al. [41]	90, Obesity: 23 pairs of twins (7-16y), including 12 male pairs	Peripheral blood samples	Weight, waist circumference (WC)	cg05684382 (chr: 12) and cg26188191 (chr: 16)	817471 qualified CpG loci; No positive sites
Li et al. [42]	30 twin pairs (15 male and 15 female pairs)	Whole blood samples	BMI	-	136; No CpGs reached genome-wide significance
Demerath et al. [43]	2097, 2377, 991, 648	Leukocyte DNA, Whole-blood DNA	BMI, WC	*HIF3A, CPT1A, ABCG1, LGALS3BP, KDM2B, PBX1, BBS2*	Probes: 76 BMI-related, 164 WC-related, 8 BMI change-related
Xu et al. [44]	510	Whole blood samples	BMI	*SOCS3*	20 (including novel cg18181703)
Sun et al. [45]	995 whites, 490 blacks; 252 whites, 228 blacks; 439 whites, 201 blacks	Whole blood samples	BMI	*PROC, PLCH1*, *SOX2OT*, *BCL9L*, *SBNO2, ABCG1*	349 (266 novel) in whites, 36 (21 novel) in blacks
Meeks et al. [46]	547	Whole blood samples	BMI, WC	*CPT1A, NLRC5, BCAT1*	18 Differentially methylated positions (DMPs) for BMI and 23 for WC, 14 DMPs overlapped between BMI and WC
Giri et al. [47]	236	Peripheral blood samples	BMI	*BAI3, SLC22A11, ZNF45*	53; 19 (BAI3), 25 (SLC22A11) and 9 (ZNF45)
Dick et al. [48]	479 339 1789	Whole-blood DNA; adipose tissue (n = 635) and skin (n = 395)	BMI	*HIF3A*	-cg22891070, cg27146050, cg16672562
Sayols-Baixeras et al. [49]	641 2,515	Blood DNA	BMI, WC	Located in 95 loci; *INTU, SFRP2, RUNX2, CTTNBP2, CPT1A, CACNA1C, MCF2L, BCL2L2, MPG, KRT16*	94 (BMI, 70 novel) and 49 (WC, 33 novel)
Mendelson et al. [50]	3,743; replication in 3 external cohorts of 4,055 participants	Whole-blood-derived DNA	BMI	*SREBF1*	83
Dhana et al. [51]	1,450 2,097	Leukocytes	BMI, WC	*MSI2, LARS2, BRDT, PSMD1, IFI44L, MAP1A, MAP3K5, LGALS3BP, MAP2K3, DHCR24, CPSF4L, TMEM49*	14 (BMI) and 26 (WC)
Do et al. [52]	46 studies	Blood-based DNA	BMI, WC	-	77 (BMI) and 4 (WC)
Nikpay et al. [53]	-	Blood-based DNA	BMI	*CCNL1, MAST3, SLC5A11, POMC, ADCY3, DNAJC27*	cg21178254, cg02814054, cg06028605, cg01884057
Benton et al. [54]	15 women	Subcutaneous and omental adipose tissue	BMI	*PROX1, PHACTR1, SLC22A8, SMAD3, LCAT, DNM2, LEPR, STAB1, ZNF608, HMGA1, MSRA, TUB, NRXN3, FTO, MC4R*	485,577
Dahlman et al. [55]	16 women	Abdominal subcutaneous fat cells	BMI	mapped to 3717 unique genes	8504
Rönn et al. [56]	23 healthy men	Adipose tissue	BMI	*RALBP1, HDAC4, NCOR2, TCF7L2, KCNQ1*	485,577 probes covering 99% RefSeq genes; 17,975 (7,663 genes)
Macartney-Coxson et al. [57]	681 (subcutaneous) 33 (omental)	White adipose tissue depots	BMI	*PITX2, ISL2, ATP2C2*	784 and 1129 extended regions
Keller et al. [58]	39 (Men), 66 (women)	Subcutaneous adipose tissue (SAT) and omental visceral adipose tissue (OVAT)	BMI	*HAND2, HOXC6, PPARG, SORBS2, CD36, CLDN1*	1381 differentially methylated genes (SAT vs OVAT)
Crujeiras et al. [59]	45 (obese) 8–10 (non-obese)	Subcutaneous adipose tissue and circulating leukocytes	BMI	*FGFRL1, NCAPH2, PNKD, SMAD3*	–
Turner et al. [60]	9 (young male) 5 (older adults)	Skeletal muscle tissue	-	*KIF15, DYRK2, FHL2, MRPS33, ABCA17P, HOXD10, HOXD9, HOXD8, HOXA3, HOXC9, HOXB1, HOXB3, HOXC-AS2, HOXC10, HOXC-AS3*	6–8
Prats-Puig et al. [61]	16 pregnant women (8 obesity; 8 control)	Umbilical cord tissue	BMI	*MYL6, MYH11, TNNT3, TPM2, CXCL2, NCAM1*	38
Sillanpää et al. [62]	139 47	Blood and muscle tissue	BMI Body composition	-	–
Day et al. [63]	7 obese females	Skeletal muscle	BMI	SORBS3	30
Nogues et al. [64]	30 women	Fetal and maternal sides of third-trimester placenta	BMI	*LEP, ADPN*	–
Gagné-Ouellet et al. [65]	262 mother–offspring	Placental DNA	BMI	*TFAP2E, FAM3C, FMN1, MAGI2, SKAP2, BMPR1B*	cg22593959 (lower) and cg22436429 (higher)
Daniels et al. [66]	135 Women and Infants (1–2 days)	Placenta	Weight, weight gain	*LEP*	–
Breton et al. [67]	276 mother–child dyads	Placenta	BMI	*NEGR1*	30 CpGs; cg26153364, cg23166710, cg04932878, cg1652573, cg23166710
Shrestha et al. [68]	301	Placenta	BMI, rate of gestational weight gain	*EGFL7, VEZT, MYT1L, DLX5, SLC35F3*	cg14568196, cg15339142, cg02301019 cg17918270, cg20735365, cg17451688, cg1456819
Rounge et al. [69]	50 lean, 50 heavy adolescent girls	Saliva	BMI	*MC2R, IGFBPL1, IP6K1, IGF2BP1*	100 (7 regions)
Oelsner et al. [70]	92	Saliva samples	BMI, weight gain	*FSTL1, SORCS2, NRF1, DLC1, PPARGC1B, CHN2, NXPH1*	17 (5 decreased methylation)
Dunstan et al. [71]	431 adolescents aged 10–15 years	Saliva samples	BMIz, WC, and percent body fat	*LEP, ICAM-1, CRH, LINE-1*	3
Kaufman et al. [72]	321 234	Saliva	BMI	*PCK2, CxCl10, BCAT1, HID1, PRDM16, MADD, PXDN, GALE*	10
Li et al. [73]	232	Saliva samples	BMI	-	28,587
Aslibekyan et al. [74]	991 Replicatin: 2,377, 2,097	CD4^+^ T-cells; Replicatin in whole blood samples	BMI, WC	*CPT1A, PHGDH, CD38*	8 (BMI) and 5 (WC)

The full name of the abbreviations can be found in the abbreviate table

#### DNAm in children

Huang et al. [22] studied DNAm profiles in whole blood from 78 obese children and 71 normal children matched for age and sex, respectively. By comparing the methylation profiles between the two groups of children, the investigators identified 129 differentially methylated CpG (DMCpG) loci and the methylation of these genes differed by > 10%. Signaling pathways with high DMCpG enrichment include immune system regulation, cell signaling regulation, developmental processes, and small GTPase-mediated signaling. In individual subjects, sodium bisulfite pyrosequencing was used to verify the association of specific DMCpG methylation with childhood obesity, such as the FYN oncogene related to SRC, FGR, YES; GALE, UDP-galactose-4-epimerase (*FYN*), Piwi-like RNA-mediated gene silencing 4 (*PIWIL4*), and thousand-and-one amino acid kinase 3 (*TAOK3*). In obese individuals, three CpG loci in *FYN* were hypermethylated, while obesity was significantly associated with hypomethylation of the CpG loci in *TAOK3* and *PIWIL4*. Each 1% increase in *TAOK3* methylation was associated with a 0.91-fold decrease in the odds of obesity, while a 1% increase in *FYN* CpG3 methylation was associated with a 1.03-fold increase in the odds of obesity [22]. These findings provide ample evidence that childhood obesity is associated with the level of specific DNAm in whole blood and that some DNAm may serve as biomarkers of the risk of obesity in the future. Fradin et al. [23] also investigated DNAm profiles of 40 obese children and controls, and identified 31 distinct methylated CpG loci in obese children. The majority of these CpG loci were hypermethylated in obesity. In severely obese children, 151 differentially methylated CpG loci were identified, 10 of which differed by more than 10% methylation. The main pathways enriched at the identified CpG loci include “insulin receptor substrate 1 (*IRS1*) target genes” and several cancer pathways [23]. This research may contribute to understand obesity and its associated complications. In 374 preschool children, 212, 230, 120, 24 and 15 specific DNAm variants were identified in whole blood samples, and they were associated with body mass index (BMI), fat-mass, fat-free-mass, fat-mass-index and fat-free-mass-index, respectively. These DNAm sites were also significantly associated with probes in genes of non-coding RNAs (ncRNAs) LOC101929268, myeloid/lymphoid or mixed-lineage leukaemia, translocated to 4 (*MLLT4*), cartilage intermediate layer protein 2 (*CILP2*), chromosome 3 open reading frame 70 (*C3orf70*), ST6 beta-galactosamide alpha-2,6-sialyltranferase 1 (*ST6GAL1*), Zinc finger protein 643 (*ZNF643*/*ZFP69B*), SOS Ras/Rac guanine nucleotide exchange factor 1 (*SOS1*), previously PR domain containing 14 (*PRDM14*), PR/SET domain 14*,* CASP8 and FADD like apoptosis regulator (*CFLAR*), extracellular leucine-rich repeat and fibronectin type III domain containing 2 (*CYTH4-ELFN2*), cytohesin 4, WD repeat domain 51A/centriolar protein A (*WDR51A*/*POC1A*), Kelch like family member 6 (*KLHL6*) and Sushi, nidogen and EGF like domains/insulin-responsive sequence DNA-binding protein 1 (*SNED1*/*IRE-BP1*) after Bonferroni correction [23]. These findings support the potential epigenetic modifications in body composition and obesity [24]. Li et al. [25] identified 226 methylated CpG loci differed between 3 obese children (mean BMI: 21.67) and 4 age/sex matched controls (mean BMI: 14.92). These methylated CpG sites were mainly engaged in immunity and lipoprotein metabolism. Then, the candidate CpG sites within the histone deacetylase 4 (*HDAC4*), regulator of axillary meristems 2 (*RAX2*), apolipoprotein A5 (*APOA5*), carboxylesterase 1 (*CES1*) and solute carrier family 25, member 20 (*SLC25A20*) genes were validated using bisulfite sequencing PCR in a cohort of 42 controls and 39 obese cases. The results revealed that methylation levels within the *RAX2* and *HDAC4* loci were positively associated, whereas those within the *CES1* and *APOA5* loci were negatively associated with childhood obesity [25]. Thus, changes in the specific CpG loci may contribute to obesity, which may provide a new direction for the etiology of obesity. Sambras et al. [26] analyzed the potential association between DNAm and childhood obesity. DNA samples of 24 children in white blood cells were obtained from the Grupo Navarro de Obesidad Infantil (GENOI) study (obesity and control groups, *n* = 12; respectively). The association between the microarray data of two CpG loci and obesity were validated in 91 children. A total of 734 CpGs (783 genes) differentially methylated were identified between the two groups. These genes were mainly enriched in circadian and oxidative stress signaling pathways. Furthermore, DNAm levels in several genes such as protein tyrosine phosphatase receptor type S (*PTPRS*), period3 (*PER3*), adenylate cyclase activating polypeptide receptor 1 (*ADCYAP1R1)*, N-methyl-D-aspartate receptor subtype 2D (*GRIN2D*), glutamate receptor, ionotropic, and vasoactive intestinal peptide receptor 2 (*VIPR2*) were associated with obesity traits. Significant correlations between methylation levels of CpG sites on *PTPRS* and *PER3* with BMIz were also identified by Sequenom MassArray technique from the replication population (*n* = 91, *r* = -0.238, *P* = 0.011 for CpG3 *PTPRS*, *r* = -0.198, *P* = 0.029 for CpG6 *PTPRS*, and *r* = -0.280, *P* = 0.004 for CpG1 *PER3*) [26].

#### DNAm in adolescents

Obesity is associated with a higher risk of cardio-metabolic diseases even in childhood and adolescence, but it is unknown whether this association is mediated by epigenetic mechanisms. Huang et al. [27] found that mid-childhood BMIz (mean age 7.7 years) was associated with cardio-metabolic risk score in early adolescence (mean age 12.9 years) via mid-childhood DNAm. DNAm in leukocytes was measured among 265 children in the Project Viva. In a high-dimensional mediation analysis and a natural effect model, an association between mid-childhood BMIz and cardio-metabolic risk score in early adolescence was observed at 6 CpG loci (natural effect model: β = 0.04, *P* = 0.032, accounting for 13% of the total effect). The natural direct effect of BMIz on cardio-metabolic risk score remained significant (β = 0.27, *P* = 1.1E-25). In addition, there were also 5 CpG sites in the opposite direction from the total effect (natural effect model: β = -0.04, *P* = 0.02) [27]. Mediation in different directions implies a complex role of DNAm in the association between BMI and cardio-metabolic risk in the childhood and adolescence. In the population-based Penn State Child Cohort follow-up exam, the epigenome-wide single nucleotide resolution of DNAm in the CpG loci and surrounding regions of 263 adolescents was obtained from peripheral leukocytes. Among the 5669 loci related to the BMI percentile with the screening rule of *P* < 0.05, 28 were identified within genes related to obesity. Obesity-related genes were significantly enriched among 103,466 intragenic loci. Furthermore, increased methylation at one locus within single-minded homolog 1 (*SIM1*) was significantly related to higher BMI percentile [28]. These data might suggest that DNAm may be related to risk of obesity in adolescents. In peripheral blood DNAm of the leptin gene (*LEP*) from the Isle of Wight Birth Cohort, 23 CpG loci were obtained in 10 years of age (*n* = 297) and 16 CpG loci in 18 years of age (*n* = 305) samples. The duration of total and exclusive breastfeeding was associated with DNAm in 4 *LEP* CpG loci at 10 years, and not at 18 years. Differential methylation region (DMR) analysis identified 5 significant differentially methylated regions. Breastfeeding duration was associated with *LEP* methylation and BMI trajectory. One DNAm loci of *LEP* methylation was associated with an early transient overweight trajectory and the other loci were associated with an early persistent obesity trajectory [29]. Furthermore, a total of 174 candidate CpG loci from whole blood DNAm of 325 subjects were screened, which also included correction loci previously found to be associated with BMI in children and adults. Of these 174 CpG loci, 38 CpG loci in prepubertal DNAm were associated with shifts in BMI status, including 30 CpG loci that were sex-specific [30]. Prepubertal DNAm was associated with changes in BMI status during pubertal development, and these associations may be gender-specific. Vehmeijer et al. [31] performed a meta-analysis of EWAS encompassing 4133 children from 23 studies to explore the association between DNAm in umbilical cord blood and whole blood and BMI in children aged 2 to 18 years. DNAm at three CpG loci (cg05937453, cg25212453, and cg10040131) of different age was associated with Bonferroni-significant BMI. DNAm at 9 additional CpG loci in the cross-sectional childhood model was associated with BMI with significance of FDR. The strength of association between DNAm and adult BMI for the 187 CpG loci previously identified was positively correlated with age in children. Additionally, the correlation coefficients between the estimates of the effect of these CpG loci in adults and children increased. The enrichment of CpG loci was also found to be higher in adults in all age groups [31].

#### DNAm in youth

Xu et al. [32] analyzed genome-wide methylation profiles of more than 470,000 CpG loci in peripheral blood samples from obese and lean (48 each) African-American youth aged 14–20 years. A large number of differentially variable CpG loci (DVC) and differentially methylated CpG loci (DMC) were identified. DVC often exhibit abnormal structures and were more variable in cases. Both DVC and DMC determined from the first group independently predicted obesity status in the second group. Furthermore, both DMC and DVC-containing genes showed significant enrichment in genes identified by GWAS for obesity and related diseases (eg, hypertension, dyslipidemia, and T2D) [32]. These findings suggest that different variants are an important feature of obesity-associated methylation. A total of 76 CpG loci associated with obesity were identified in the EWAS study of 700 African Americans aged 14–36 years. Fifty-four of these loci were validated in the Atherosclerosis Risk in Communities (ARIC) study of 2097 African Americans aged 47–70 years, 29 of which were newly associated with obesity. Thirty-seven CpG loci were replicated in neutrophils, and 51 CpG loci were associated with at least one cardiometabolic risk factor. However, the number of CpG was reduced to 9 after adjustment for obesity. Seventeen CpG loci were associated with the expression of 17 genes in the cis, and five of which were expressed at different levels between obese and lean individuals [33].

#### DNAm in women

Wilson et al. [34] performed a genome-wide analysis of DNAm and BMI using data from a subset of women in a Sister Study. Blood DNAm data were obtained from 871 women. The associations were replicated in a non-overlapping group of 187 women from Sister Studies. Four CpG loci in the discovery set were significantly different, and 5 CpG loci were replicated by pyrosequencing; 4 CpG loci in the replication set were significantly different in the Bonferroni-correction statistics, 23 loci passed FDR. Several genes have been associated with obesity and obesity-related chronic diseases, including angiopoietin 4 (*ANGPT4*), RAR-related orphan receptor C (*RORC*), suppressor of cytokine signaling 3 (*SOCS3*), serine/threonine kinase 39 (*STK39*), fibronectin type III and SPRY domain containing 2 (*FSD2*), xylosyltransferase 1 (*XYLT1*), ATP binding cassette subfamily G member 1 (*ABCG1*), ankyrin repeat and SOCS box containing 2 (*ASB2*), and corticotropin releasing hormone receptor 2 (*CRHR2*) [34]. Leukocyte EWAS testing was performed on 60 young women each in obese and lean groups. It was also validated in obesity and monozygotic twins. The results revealed that both populations at both adolescence and adulthood, with obese individuals having a higher proportion of differential hypomethylation. EWAS found significant differences in cytochrome C oxidase subunit 6A1 pseudogene 2/faciogenital dysplasia 2 (*COX6A1P2*/*FGD2*), strawberry notch homolog 2 (*SBNO2*), testis expressed 41 (*TEX41*), ribosomal protein S6 kinase A2 (*RPS6KA2*), DNA methyltransferase 1-associated protein (*DMAP1*), *SOCS3* and SET binding protein 1 (*SETBP1*) and other genes associated with enhancer loci [35]. Recently, Shen et al. [36] evaluated hypoxia-inducible factor 3α subunit (*HIF3A*) methylation levels in 927 individuals in the Mexican American Cohort study and identified 3 high levels of CpG loci in obese women: 46801699, 46801642, and 46801557, and the methylation levels at these 3 CpG loci were associated with body weight [36]. These results provide evidence supporting the role of *HIF3A* in obesity and weight gain.

#### DNAm in men

Guay et al. [37] verified whether DNAm of the adrenoceptor beta 3 (*ADRB3*) in blood and visceral adipose tissue is associated with obesity and its related complications. DNAm levels were measured in 61 men with familial hypercholesterolemia (FH) and 30 men with severe obesity, and common *ADRB3* polymorphisms were genotyped. The results revealed that *ADRB3* DNAm levels were negatively correlated with low-density lipoprotein cholesterol (LDL-C) levels in FH, and waist-to-hip ratio in severely obese men [37]. These results suggest that epigenetic changes in *ADRB3* may be involved in the development of obesity and its associated metabolic complications. Skuladottir et al. [38] investigated the effect of total sleep deprivation (TSD) on the 5'-prime region of stearoyl-CoA desaturase 1 (*SCD1*) DNAm. A total of 16 healthy young men participated in the study, and subjects received one night of TSD and normal sleep, and fasting whole blood samples were collected the following morning for analysis. Three CpG loci (cg14089512, cg24503796 and cg00954566) closed to the *SCD1* transcription start loci were found to have significant methylation differences. The levels of both *SCD-16* and *SCD-18* were significantly high after overnight TSD and were significantly correlated with changes in the 3 loci mentioned above [38]. These results suggest a close relationship between epigenetic alteration and TSD, *SCD1* expression, and fatty acid synthesis. Keller et al. [39] peformed a genome-wide DNAm analysis of 120 subjects who were on a Mediterranean/low-carbohydrate or low-fat diet for 18 months, comparing male subjects with the most significant weight loss and gain, and identified significant changes in DNAm for several genes, including leucine rich repeat containing 27 (*LRRC27*), cysteine rich secretory protein 2 (*CRISP2*), and schlafen family member 12 (*SLFN12*). These genes are mainly enriched in biological processes such as cell adhesion and calcium ion binding. Fifteen CpG loci were negatively associated with weight change, including nudix hydrolase 3 (*NUDT3*) and nuclear receptor corepressor 2 (*NCOR2*). Baseline DNAm scores better predict successful weight loss compared to age and BMI. Additionally, identifying methylation differences in certain genes can be used as prognostic biomarkers for obesity [39].

#### DNAm in twins

Monozygotic twins with discordant BMI are ideal subjects for the analysis of epigenetic associations between DNAm and BMI, with perfect control for their genetic makeup, primarily responsible for BMI variation. Ollikainen et al. [40] performed a genome-wide leukocyte DNAm variation in 30 pairs of clinically healthy young adult monozygotic twins with discordant BMI (mean within-pair difference: 5.4 ± 2.0 kg/m^2^). No differentially methylated CpG loci were detected between BMI-discordant twins. However, twin stratification based on liver fat accumulation levels revealed two highly epigenetically distinct groups. Significant DNAm differences between twins (*n* = 1236 CpG loci) were observed only when the heavier twins had excess liver fat (*n* = 13 twins). This unhealthy pattern of obesity is strongly associated with insulin resistance and hypo-inflammation. The differentially methylated CpG loci included 23 genes known to be associated with obesity, adiposity, T2D and MetS, as well as potentially novel metabolic genes (*ATP10A*, *BCAS3*, *BCL2*, *CDKAL1*, *CMIP*, *CTSZ*, *ETV5*, *FBP2*, *GGT1*, *HHEX*, *HK1*, *KCNQ1*, *LGALS2*, *MAP2K5*, *NR1H3*, *PNOC*, *PRDM2*, *RPTOR*, *SLC39A12*, *ST3GAL4*, *THADA*, *TNNI3K* and *UBASH3A*). Furthermore, a significant clustering of differentially methylated genes was observed in the metabolic pathways of vitamin, amino acid, fatty acid, sulfur and renin-angiotensin [40]. These findings suggested that several novel candidate genes and pathways were associated with obesity and obesity-related complications. Li et al. [41] explored the DNAm loci based on discordant monozygotic twins in 2016, 90 pairs of twins aged 6–17 years were recruited in Beijing, and 23 of these twins (12 pairs male) were tested for DNAm and a total of 817,471 eligible CpG loci were included in the genome-wide association analysis. No positive loci associated with obesity were found. However, the results indicated that loci cg05684382 and cg26188191 may play a role in obesity [41]. In another EWAS [42], a total of 15 male and 15 female identical twins aged between 39 and 72 years participated (BMI: 3–7.5 kg/m^2^) in the study. No CpG loci had genome-wide significance. Genomic region-based analysis identified 11 differentially methylated regions comprising coding and non-coding genes, some of them were validated by gene expression analysis of independent samples. DNAm sequencing analysis of identical twins provides new insights into the epigenetic regulation of BMI and obesity [42].

#### DNAm in different races

##### African–American

Leukocyte DNAm analysis was performed in 2097 African American adults in the ARIC study cohort and validated in 2377 whites in the Framingham Heart Study cohort. CD4^+^ T cell assays were performed in 991 Whites in the Genetics of Lipid Lowering Drugs and Diet Network Study cohort and were tested using adipose tissue DNA from 648 women in the Multiple Tissue Human Expression Resource cohort. These studies identified 76, 164, and 8 probes associated with BMI, waist circumference (WC), and BMI changes, respectively. The probes included the recently reported *HIF3A*, carnitine palmitoyltransferase 1A (*CPT1A*) and *ABCG1,* and 1 additional WC probe achieved replication using blood DNA, 16 of which also replicated in adipose tissue, including those involved in lipid metabolism, immune response/cytokine signaling and other diverse pathways. Furthermore, 15 new methylation loci were also identified, including the galectin 3 binding protein (*LGALS3BP*), lysine demethylase 2B (*KDM2B*), PBX homeobox 1 (*PBX1*) and Bardet-Biedl Syndrome 2 (*BBS2*), and these loci were overlapped in different studies [43]. Xu et al. [44] performed a DNAm association analysis of obesity at a community center in New Haven, CT, USA, and investigated the effect of cumulative stress on DNAm and body weight. Twenty BMI-related CpG loci were identified, including a novel locus cg18181703 for *SOCS3*. The interaction between this novel locus and cumulative stress jointly altered body weight. Individuals with at least five significant life events and low levels of methylation of cg1818703 exhibited a 1.38-fold risk of obesity [44]. These findings suggest that DNAm abnormalities are associated with body weight and that *SOCS3* methylation moderates the effects of cumulative stress on obesity. Sun et al. [45] performed a race-specific EWAS in whites and blacks from the Bogalusa Heart Study and the Georgia Stress and Heart Study, examined twice over 6.2 years, and analyzed the temporal relationship between DNAm and BMI. Finally, 349 CpG loci (266 novel) in whites and 36 CpG loci (21 novel) in blacks were associated with BMI, and 8 (1 novel) CpG loci overlapped between black and white races. A total of 18 CpG loci in whites and 7 in blacks had significant unidirectional paths. Baseline BMI was associated with DNAm scores (in both blacks and whites) [45].

##### Sub-Saharan Africans

Meeks et al. [46] performed EWAS on whole blood samples from 547 Ghanaians in the study of Research on Obesity and Diabetes among African Migrants (RODAM) study. They identified 18 differentially methylated positions (DMPs) for BMI and 23 DMPs for WC, of which 3 were for obesity, 1 for abdominal obesity, and 14 DMPs overlapped between BMI and WC. The DMP cg00574958 annotated as *CPT1A* was the only DMP associated with all analysed results, accounting for 6.1% and 5.6% of obesity and abdominal obesity, respectively. Nucleotide-binding oligomerization domain-like receptor family caspase recruitment domain containing 5 (*NLRC5*) cg07839457 and branched chain amino acid transaminase 1 (*BCAT1*) cg20399616 were significantly associated with BMI, obesity and WC [46].

##### Indian

Giri et al. [47] performed a two-staged GWAS of BMI in 5973 Indian adults and further replicated the main findings in 1286 Indian adolescents. This study found that the new association of rs6913677 variant in ABI family member 3 /Adhesion G protein-coupled receptor B3 (*BAI3*/*ADGRB3*) and rs2078267 variant in solute carrier family 22 member 11 (*SLC22A11*) was of GWAS significance, while rs8100011 in zinc finger protein 45 (*ZNF45*) was near GWAS significance. Variants in *BAI3* and *SLC22A11* were found to determine the methylation patterns of specific CpG loci, which contain key cis-regulatory elements. Furthermore, the *BAI3, SLC22A11,* and *ZNF45* variants were located in the suppressor chromatin, active enhancer, and active chromatin regions of human subcutaneous adipose tissue in the ENCODE database, respectively. In addition, these genomic regions are potential binding loci for the core transcription factors associated with obesity and/or metabolic disorders. Interestingly, the Genotype-Tissue Expression portal identified rs8100011 as a strong quantitative trait locus for cis-expression, while the expression of *ZNF45* in Indian skeletal muscle was negatively correlated with BMI, suggesting a possible role in obesity. This study identified three novel population-specific functional gene variants (rs6913677, rs8100011, and rs2078267) in two novel genes (*SLC22A11* and *ZNF45*) and one previously reported gene (*BAI3*) [47]. These loci may become targets for drug therapy in the future.

##### European

Dick et al. [48] performed a genome-wide analysis of methylation at the CpG loci associated with BMI. A total of 479 persons of European ancestry participated and the association of methylation levels and BMI was tested. The methylation loci associated with BMI were replicated in another cohort. Significant loci were selected for testing in a second cohort of 1789 white patients from the Cooperative Health Research in the Region of Augsburg (KORA) cohort. Five probes that encompassed three genes were found to deactivate the associated BMI in the DNA of adipose tissue (*n* = 635) and skin (*n* = 395) of white women participating in the MuTHER study. Three of these probes—cg22891070, cg27146050, and cg16672562 are *HIF3A* introns, and their association was confirmed in both cohorts. Each 0.1 increase in the beta value of methylation of cg22891070 was associated with a 3.6% (95% CI: 2.4–4.9) increase in BMI, and in the first replication cohort with a 2.7% increase and 0.8% in the second cohort. For the MuTHER cohort, methylation of cg22891070 was only associated with BMI in adipose tissue, but not in skin. A significant negative correlation was observed between the methylation of cg22891070 and the expression of a *HIF3A* gene expression probe in adipose tissue (*P* = 0.005). An increased BMI in adults of European ancestry was associated with an increase in the methylation of the *HIF3A* locus in blood cells and adipose tissue [48]. These findings suggest that perturbation of the hypoxia-inducible transcription factor pathway may play an important role in the response to gain body weight. Sayols-Baixeras et al. [49] designed an EWAS with a discovery phase in a subsample of 641 REGICOR study participants and validated with an analysis of 2515 participants in the Framingham Offspring Study. A total of 94 CpG loci were associated with BMI, and 49 CpG loci were associated with WC at 95 loci. Of these, 70 BMI-related CpG loci and 33 WC-related CpG loci were newly discovered. These CpG loci explained 25.94% and 29.22% of the variability of BMI and WC in the REGICOR samples, respectively. They also evaluated 65 of 95 validated loci in the GIANT genome-wide association data; 10 of them had Tag SNPs associated with BMI [49]. Mendelson et al. [50] performed an association study of BMI and methylation loci by microarray detection of more than 400,000 CpG loci in 3743 participants in the Framingham Heart Study and the Lothian Birth Cohorts. The independent replication studies were then carried out in 3 additional cohorts totaling 4055 participants. They identified novel differential methylation at 83 CpG loci that were associated with gene expression in lipid metabolism pathways. Genetic instrumental variable analysis of altered methylation at one of the 83 replicated CpG loci, cg11024682 (intronic to sterol regulatory element binding transcription factor 1, *SREBF1*), was associated with BMI, adiposity-related traits, and coronary artery disease. A significant proportion of methylation (16 of 83) was found to be secondary to differences in BMI [50]. These findings suggest that BMI is strongly associated with differential methylation and also provide new insights into the pathogenesis of obesity and its associated diseases. Dhana et al. [51] conducted an EWAS on obesity-related traits. Data from the Rotterdam Study (RS, discovery panel) and the ARIC Study (replication panel) were used. Fourteen CpG loci in RS were associated with BMI and 26 CpG loci with WC, of which 12 and 13 CpG loci were replicated in the ARIC study, respectively. The most important novel CpG loci were located in Musashi RNA binding protein 2 (*MSI2*, cg21139312) and Leucyl-TRNA synthetase 2, mitochondrial (*LARS2*, cg18030453). The CpG loci of the Bromodomain testis associated (*BRDT*), proteasome 26S subunit, non-atpase 1 (*PSMD1*), interferon induced protein 44 like (*IFI44L*), microtubule-associated protein 1A (*MAP1A*), and mitogen-activated protein kinase kinasekinase 5 (*MAP3K5*) were associated with BMI. CpG loci at *LGALS3BP*, mitogen-activated protein kinase kinase 3 (*MAP2K3*), 24-dehydrocholesterol reductase (*DHCR24*), cleavage and polyadenylation specific factor 4 (*CPSF4L*), and transmembrane protein 49 /vacuole membrane protein 1 (*TMEM49*/*VMP1*) were associated with WC [51]. These results provide further details of the obesity-related features, which can help identify new biomarkers in chronic diseases related to obesity.

#### Meta-analysis of blood-based DNAm

A meta-analysis is an analytical review of multiple studies on a common topic. It can be performed in any field of study where there are a number of statistical research literatures. Although this type of analysis has advantages, it also has disadvantages, such as selection bias and statistical distortions that can lead to erroneous conclusions. Do et al. [52] systematically searched 4 databases for studies published in January 2020 on obesity associated with genome-wide DNAm in non-pregnant adults aged 18–75 years. Eligible studies included cross-sectional, longitudinal, or intervention studies. A weighted sum of the Z-score meta-analysis of blood-based DNAm results with BMI and WC was performed. Forty-six out of 10,548 studies were included in systematic review, and 18 and 9 studies were included in meta-analyses of BMI and WC, respectively. In blood samples, the 77 and 4 CpG loci were significant in more than two studies of BMI and WC, respectively. Fifty-two CpG loci were significantly associated with BMI using a genome-wide significance threshold. These loci have also previously been associated with diseases such as T2D, cardiovascular disease, Crohn’s disease, and depression [51]. This meta-analysis suggests that DNAm at 52 CpG loci represents potential targets for chronic diseases associated with obesity and may be novel targets for intervention or treatment. More recently, Nikpay et al. [53] also searched genome-wide for obesity-associated methylation loci and tested whether these loci have an effect on BMI by Mendelian randomization. The study found that multiple methylation loci were associated with the risk of obesity. Low-level methylation of the cg21178254 locus upstream of cyclin L1 (*CCNL1*) increased the expression of this gene and promoted obesity. The high-level of the cg02814054 locus increased the risk of obesity by reducing the expression of microtubule associated serine/threonine kinase 3 (*MAST3*), while low-level methylation of cg06028605 increased the risk of obesity by decreasing the expression of solute carrier family 5 member 11 (*SLC5A11*). A rare variant in 2p23.3 decreased proopiomelanocortin (*POMC*), adenylate cyclase 3 (*ADCY3*) and DnaJ heat shock protein family (Hsp40) member C27 (*DNAJC27*) by promoting methylation at the cg01884057 locus expression [53]. This study reveals how specific methylation loci are functional and explains the mechanisms by which rare methylation causes disease.

### DNAm in adipose tissues

Rönn et al. [16] analyzed DNAm at approximately 480 000 loci in adipose tissue of 96 men and 94 women, as well as methylation related to age, BMI, and glycosylated hemoglobin, and compared adipose tissue and epigenetic signatures in blood. Age was significantly associated with DNAm alterations and expression of 1050 genes including four and a half LIM domains 2 (*FHL2*), NADPH oxidase 4 (*NOX4*), and plasminogen (*PLG*). Interestingly, several reported epigenetic biomarkers also showed a significant correlation between DNAm and age in adipose tissue, such as ELOVL fatty acid elongase 2 (*ELOVL2*), *FHL2*, Kruppel like factor 14 (*KLF14*), and glycine receptor alpha 1 (*GLRA1*). They identified 2825 genes, such as FTO alpha-ketoglutarate dependent dioxygenase (*FTO*), inter-alpha-trypsin inhibitor heavy chain 5 (*ITIH5*), C–C motif chemokine ligand 18 (*CCL18*), mitochondrial carrier 2 (*MTCH2*), *IRS1*, and secreted phosphoprotein 1 (*SPP1*) in which both DNAm and expression were correlated with BMI. Pathway analysis revealed that age- and BMI-related methylation levels account for a large proportion of genes associated with cancer, T2D, and cardiovascular disease [16]. These results highlight the impact of BMI on epigenetic variation in candidate genes for obesity in human adipose tissue. Epigenetic biomarkers in blood can reflect age-related epigenetic characteristics in tissues targeted for metabolic diseases, such as adipose tissue. Benton et al. [54] investigated DNAm in adipose tissue from obese women before and after gastric bypass and significant weight loss. A total of 485,577 CpG loci were analyzed. This study showed significant differences in the levels of methylation in adipose tissue. Many CpG loci (3' untranslated region and gene bodies were more frequent) had high levels of methylation before weight loss. Differential methylation was found in genes associated with obesity, epigenetic regulation and development, such as cholesteryl ester transfer protein (*CETP*), forkhead box P2 (*FOXP2*), *HDAC4*, *DNMT3B*, potassium voltage-gated channel subfamily Q member 1 (*KCNQ1*) and homeobox (*HOX*) clusters. It was also found that the mean DNAm extent of all CpG loci was lower in post-obese adipocytes compared to non-obese women [54]. In a previous DNAm analysis conducted by Dahlman et al. [55], the mean levels of DNAm of all analyzed CpG loci were lower in adipocytes in obese individuals. A total of 8504 CpG loci were differentially methylated (FDR 1%). Differentially methylated DNA loci (DML) were under-represented on CpG islands and surrounding shores. The 8504 DML mapped to 3717 unique genes that were over-represented in cell differentiation pathways. In particular, 27% of genes related to adipogenesis showed DML in obese versus non-obese women. They also explored DNAm and expression of genes related to adipogenesis in more detail in adipose tissue samples. DML annotated to adipogenesis genes was not accompanied by differential gene expression. In contrast, obese women had differential expression changes in adipogenesis genes [55]. Hypomethylation of CpG and over-representation of DML may promote adipose hyperplasia. Rönn et al. [56] reported the genome-wide pattern of DNAm in human adipose tissue before and after a 6-month exercise intervention in 23 healthy men with lower prior levels of physical activity. Differences in DNAm in adipose tissue were also studied between 31 individuals with and without a family history of T2D. The global DNAm was changed after the exercise intervention, and changes in DNAm levels were observed at 17975 CpG loci assigned to 7663 unique genes. Differential mRNA expression was present in 1/3 DNAm altered gene regions, including ralA binding protein 1 (*RALBP1*), *HDAC4* and *NCOR2*. Increased DNAm in the *RALBP1* promoter in vitro inhibited the transcriptional process. Furthermore, 18 obesity and 21 candidate genes for T2D had different CpG loci that responded to exercise in adipose tissue DNAm, including transcription factor 7 like 2 (*TCF7L2*) and *KCNQ1*. The mRNA expression of these six genes changed simultaneously. To understand whether genes with different DNAm and mRNA status affect adipocyte metabolism, investigators silenced *HDAC4* and *NCOR2* genes in 3T3-L1 adipocytes, respectively, and found increased adipogenesis in both the basal and insulin-stimulated states, respectively [56]. These findings suggest that exercise-induced changes in adipose tissue DNAm may affect cellular metabolism. Macartney-Coxson et al. [57] performed a study similar to Benton et al. [54]. They observed 3239 and 7722 differentially methylated CpG loci, including 784 and 1129 extended regions, respectively, most of which were concordant at the time point, with genes enriched in transcriptional regulation and/or development (e.g. homeobox genes). Other differentially methylated loci were observed at one time point. Investigators observed strong correlations between DNAm and clinical characteristics, particularly for CpG sites within paired like homeodomain 2 (*PITX2*) and fasting glucose, and 4 CpG sites within ISL lim homeobox 2 (*ISL2*) and high-density lipoprotein. A single CpG locus (cg00838040, ATPase secretory pathway Ca^2+^ transporting 2, *ATP2C2*) had strong tissue separation and was validated in subcutaneous and omental lipids [57]. Keller et al. [58] examined the relationship between obesity and genome-wide DNA promoter methylation and mRNA profiles in subcutaneous adipose tissue and visceral omental adipose tissue and found a negative association between methylation and several obesity-related genes and replicated ETV6 in 2 independent cohorts. In addition, six adipose tissue depot-specific genes were identified, including claudin 1 (*CLDN1*), cluster Determinant 36 (*CD36*), sorbin and SH3 domain containing 2 (*SORBS2*), peroxisome proliferator activated receptor gamma (*PPARG*), homeobox C6 (*HOXC6*), and heart and neural crest derivatives expressed 2 (*HAND2*) [58]. These findings suggest that tissue-specific epigenetic alterations in different tissues are associated with obesity. Crujeiras et al. [59] isolated DNA samples from subcutaneous adipose tissue and circulating leukocytes to reflect the specific DNAm status of adipose tissue. Comparisons by Wilcoxon ranking tests revealed global hypomethylation of differentially methylated CpG loci in obese subcutaneous adipose tissue and leukocytes. The overlap analysis yielded many genes mapped by common differentially methylated CpG loci that were identified to reflect obesity status in leukocytes, including the fibroblast growth factor receptor like 1 (*FGFRL1*), non-SMC condensin II complex subunit H2 (*NCAPH2*), PNKD metallo-beta-lactamase domain containing (*PNKD*), and SMAD family member 3 (*SMAD3*) [59]. Therefore, this research provides a new and valuable biomarker of obesity-related adipose tissue by peripheral blood analysis.

### DNAm in skeletal muscles

Turner et al. [60] compared the methylation degree between young and old human skeletal muscle and muscle-derived human primary cells (HDMCs) at different differentiate on time points (0, 1, 7, and 10 days). Aged muscle tissue was hypermethylated and reduced MyoD/Myogenin gene expression compared to young cells. Although young cells showed little change in DNAm during differentiation, aged cells exhibited extensive and marked DNAm changes in focal adhesion and PI3K-AKT signalling pathways, especially at 7 days of differentiation. Most notably, differential methylation analysis of chromosomal regions identified three locations. These locations enriched for 6–8 CpG loci in the *HOX* family (*HOXD1, 8, 9, HOXA3, HOXC9, HOXB1, 3, HOXC-AS2* and *HOXC10*) were all hypermethylated in aged tissue. Aged cells showed the most variable methylation at day 7, with hypermethylated *HOXC-AS3, HOXB1, HOXC9* and *HOXD8* and hypomethylated *HOXC-AS2* and *HOXC10*. There was a negative correlation between DNAm and gene expression of *HOXB1, HOXA3,* and *HOXC-AS3*. Compared to aged humans, young adults increased physical activity and had an inverse regulation of *HOXB1* and *HOXA3* methylation [60]. These findings suggest that HOX genes are subject to differential epigenetic regulation in skeletal muscle and HDMCs in aged humans, and increased physical activity may help prevent these changes. Prats-Puig et al. [61] performed DNAm measurements in 16 pregnant women (8 each for obese subjects and controls) to investigate whether gestational obesity was associated with DNAm changes in skeletal muscle-specific genes in umbilical cord tissue. Identification analysis reported 38 differentially methylated CpG loci in four skeletal muscle-specific genes: contractility, structure, actin, and myogenesis. Compared to controls, obesity during pregnancy resulted in higher hypermethylation and lower hypomethylation. Hypermethylation of CpG loci was close to transcription loci and with high CpG density, and hypomethylation of regions was away from transcription loci and with low CpG density. The co-methylation network was also reduced in total interactions [61]. Sillanpää et al. [62] investigated the correspondence of different estimates of biological aging in whole blood samples and muscle samples, and their relationships to body composition, physical function, and physical activity in two independent cohorts. The age of DNAm was estimated using genome-wide methylation data and publicly available data. The R-package “Muscle Epigenetic Age Test” (MEAT) was used to estimate the methylation age of muscles. Blood and muscle DNAm age estimates were highly correlated with chronological age, but correlations between different age-accelerated estimates were weak. The muscle aging of monozygotic twins was faster, while the correlation between the estimation of age acceleration and physical activity, physical function and body composition was weak, which was mainly explained by smoking and gender. The MEAT was developed to predict chronological age, which may explain why its lack of correlation with functional phenotypes. Day et al. [63] investigated skeletal muscle DNAm of sorbin and SH3 domain containing 3 (*SORBS3*) with gastric bypass surgery (GBS). Basal muscle biopsies were obtained from 7 morbidly obese females before and three months after GBS. Thirty methylation loci were significantly altered in *SORBS3* using reduced representation bisulfite sequencing, 29 of 30 loci decreased post-GBS compared to pre-GBS. The methylation in two loci (Chr.8:22423690 and Chr.8:22423702) of the 29 decreased *SORBS3* loci. The decreased methylation was associated with an increased in *SORBS3* expression after surgery (fold change + 1.7). Furthermore, methylation of the *SORBS3* promoter significantly altered reporter gene expression in vitro. Two *SORBS3* methylation loci (Chr.8:22423111 and Chr.8:22423205) were strongly correlated with fasting blood glucose levels. Changes in *SORBS3* expression after surgery were correlated with obesity measures and fasting insulin levels [63]. These results suggest that *SORBS3* methylation and gene expression are altered in obesity and restored to normal levels by GBS-induced weight loss.

### DNAm in placenta

It is well known that an imbalance in the ratio between leptin and adiponectin is associated with obesity. A recent report found that maternal obesity had a key impact on placental development. Nogues et al. [64] characterized the placental expression and DNAm of both adipokine ligands and receptors in obese women. Tissues were collected from both the maternal and fetal sides of pregnant women in the third trimester of pregnancy for testing, respectively. The results revealed higher levels of leptin on the fetal side than on the maternal side. Among them, maternal obesity was associated with elevated leptin promoter DNAm on the fetal side and hypomethylation on the maternal side of the adiponectin promoter, low levels of leptin receptor protein, low levels of adiponectin receptor 1 transcript, and high levels of promoter DNAm of the adiponectin receptor 2 [64]. These results suggest that maternal obesity is associated with the down-regulation and epigenetic alterations of leptin/adiponectin in the placenta, providing new ideas for great understanding of the mechanisms of obesity. Gagné-Ouellet et al. [65] identified placental DNAm variation associated with adiposity at 3 years of age. Placental DNAm and mRNA levels for the annotated gene were quantified in 262 mother–offspring. Lower DNAm at cg22593959 (chr7q31.3) and cg22436429 was correlated with higher adiposity. DNAm in cg22593959 and cg22436429 was associated with mRNA levels in the FAM3 metabolism regulating signaling molecule C (*FAM3C*) and *TFAP2E*. Four genomic regions were also associated with skinfold thickness within bone morphogenetic protein receptor type 1B (*BMPR1B*), SRC kinase associated phosphoprotein 2 (*SKAP2*), WW and PDZ domain containing 2 (*MAGI2*), membrane associated guanylate kinase andformin 1 (*FMN1*). Daniels et al. [66] examined maternal diet, placenta *LEP* DNAm, and neonatal growth in a sample of healthy newborns recruited within 1–2 days and their mothers (*n* = 135). The rs2167270 was a key genotype for predicting placental *LEP* DNAm, and lower levels of *LEP* methylation were significantly associated with high intake of carbohydrates. Additionally, total caloric intake was also associated with placenta *LEP* methylation. However, the significance was not increased after controlling for relevant covariates [66]. These findings emphasize the importance of carbohydrate intake in *LEP* methylation in the placenta. Methylation decreases gene transcription, while low levels of methylation may increase the response of placenta to high caloric intake and carbohydrate. Studies have also shown that maternal pre-pregnancy obesity and hyperglycemia are associated with complications such as metabolic and neurodevelopmental in children. DNAm may adapt the fetus to a hostile environment through key gene loci. Neuronal growth regulator 1 (*NEGR1*) is involved in both energy balance and behaviour regulation. Breton et al. [67] investigated the associations between placental DNAm at the *NEGR1* locus and the anthropometric and neurodevelopmental status of preschool children. A total of 276 mother–child pairs from the Gen3G birth cohort were analyzed. DNAm levels at 30 CpG loci of the *NEGR1* locus were examined in placental biopsies. Four of these DNAm from the CpG loci in front of the second exon of *NEGR1* predicted BMIz in children. The metabolic profile of the mother during pregnancy with levels of the *NEGR1* DNAm explained 7.4% of the variation in BMIz [67]. This study suggests that placental *NEGR1* DNAm is associated with obesity and related comorbidities in preschool-aged children. Obesity before or during pregnancy affects fetal growth and puts the fetus at risk of obesity as it gets older. The epigenetic mechanisms of the placenta may be the basis for these associations. Shrestha et al. [68] conducted an EWAS to determine the relationship between placental DNAm changes and pre-pregnancy BMI and the gestational weight gain rate from the first to third trimester (GWG1-3). The database used had with genome-wide placental DNAm and gene expression data from NICHD Fetal Growth Studies. Pre-pregnancy BMI was associated with DNAm at cg14568196 (EGF like domain multiple 7, *EGFL7*), cg15339142 (vezatin, adherens junctions transmembrane protein, *VEZT*), and cg02301019 (AC092377.1). GWG1/2 were associated with DNAm at cg17918270 (myelin transcription factor 1 like, *MYT1L*), cg20735365 (distal-less homeobox 5, *DLX5*), and cg17451688 (solute carrier family 35 member F3, *SLC35F3*). Both pre-pregnancy BMI and DNAm at cg1456819 were negatively correlated with *EGFL7* expression in the placenta. Multiple CpG loci were associated with chilhood and adult prepregnancy BMI in the placenta. Functional annotation revealed that *EGFL7* was highly expressed in the placenta and that its methylated CpG loci near *EGFL7* and *VEZT* were targets of cis-meQTL in the blood [68]. These overlapping CpG loci suggest that epigenetic changes in the placenta contribute to understanding the early origins of obesity.

### DNAm in saliva

Saliva is a practical and widely available biological sample. Some experts believe that the salivary DNAm pattern of key obesogenic genes in children may be associated with BMI in mothers and can help to identify pathways associated with children at risk of obesity. Rounge et al. [69] performed an EWAS in the Finnish adolescent health cohort to determine the relationship between DNAm and adolescent BMI. Saliva samples from 50 lean and 50 obese adolescent girls were analyzed for differential DNAm at 3.1 million CpG loci. A total of 100 CpG loci with statistically significant differences were identified, including 7 “bumphunting” regions and 5 CpG islands. Ten CpG loci were closely associated with BMI, with substantial overlap with obesity and insulin-related genes, including the melanocortin 2 receptor (*MC2R*), insulin-like growth factor binding protein like 1 (*IGFBPL1*), inositol hexakisphosphate kinase 1 (*IP6K1*) and insulin like growth factor 2 MRNA binding protein 1 (*IGF2BP1*) [69]. Oelsner et al. [70] performed a genome-wide DNAm analysis of 92 saliva samples collected from Hispanic pre-school children to investigate the epigenetic patterns. The DNAm of child at 17 CpG loci was significantly associated with maternal BMI, with increased methylation at 12 CpG loci and decreased methylation at 5 CpG loci. The results of the pathway analysis suggested that methylation at the 17 CpG loci was related to methionine degradation, cysteine biosynthesis, and circadian rhythms. Furthermore, 8 of the 17 CpG loci located in the follistatin like 1 (*FSTL1*), sortilin related VPS10 domain containing receptor 2 (*SORCS2*), nuclear respiratory factor 1 (*NRF1*), DLC1 Rho GTPase activating protein (*DLC1*), PPARG coactivator 1 beta (*PPARGC1B*), chimerin 2 (*CHN2*), and neurexophilin 1 (*NXPH1*) have previously associated with obesity, diabetes, and insulin pathways [70]. These salivary findings suggest potential epigenetic differences in the risk of childhood obesity in Hispanic preschoolers. Identifying early biomarkers and understanding the underlying epigenetic changes can provide direction for early intervention in childhood obesity. Dunstan et al. [71] evaluated the associations between DNAm and anthropometric and body composition measurements. DNAm of promoter regions (3–4 CpG loci each) of the *LEP*, *ICAM-1*, *CRH*, and *LINE-1* was measured in 431 adolescents. Sex-stratified analysis after adjustment of age showed that only the 3 correlated outcomes in obese boys were inversely associated with *LEP* methylation [71]. These findings suggest that saliva could be a possible sample for epigenetic studies in adolescents. Kaufman et al. [72] determined whether measures of adverse childhood experiences and DNAm were associated with obesity indices in youth. Children (*n* = 321) aged 8 to 15 years were recruited to conduct the survey. Obesity assessments and salivary DNA were examined for 234 participants. Ten methylated loci were found to interact with adverse childhood experiences to predict BMI on cross-sectional measures. Six loci were found to play a major role on the prediction of BMI. Eight loci such as UDP-galactose-4-epimerase (*GALE*), MAP kinase activating death domain (*MADD*), PR/SET domain 16 (*PRDM16*), peroxidasin (*PXDN*), HID1 domain containing (*HID1*), *BCAT1*, C-X-C motif chemokine ligand 10 (*CxCl10*), and phosphoenolpyruvate carboxykinase 2 (*PCK2*) were associated with the risk of obesity [72]. This study sets the stage for future longitudinal studies to further help reduce the adverse effects associated with adversity. Li et al. [73] estimated cross-tissue DNAm age acceleration using saliva samples from 232 African American mothers. After adjusting for multiple confounders, each 1 kg/m^2^ increase in BMI was associated with an accelerated increase of 0.14-year in DNAm age acceleration [73]. These findings support that high BMI accelerates aging and plays a key role in age-related diseases.

## Histone modification

**Fig. 5:**
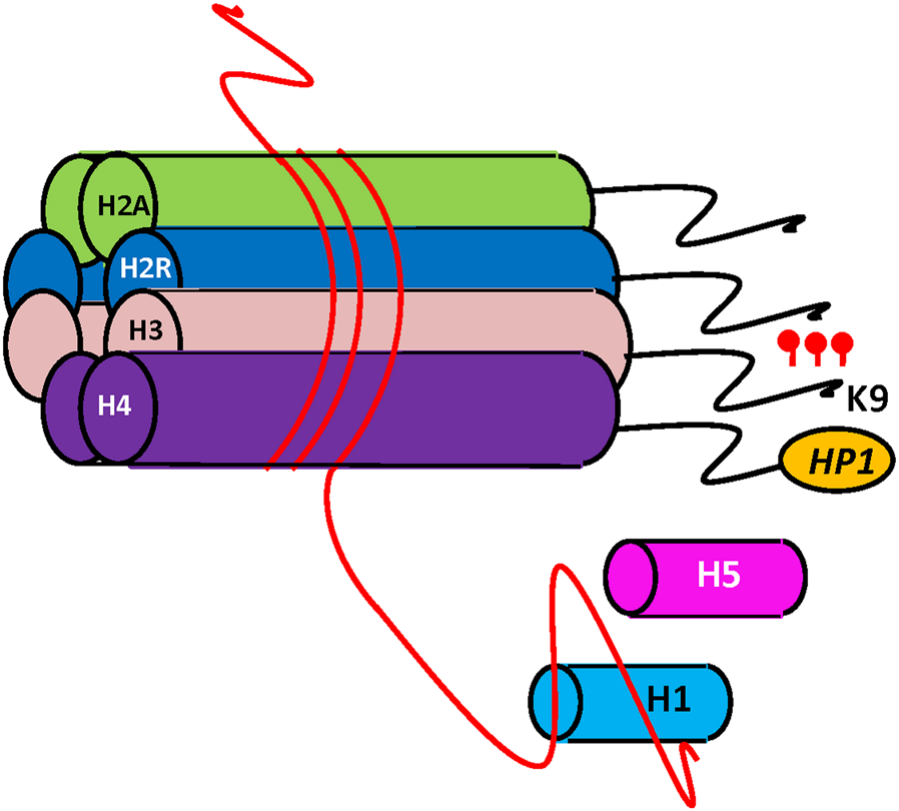
The simplified diagram of histone modification. The histone octamer “bead” was surrounded by a DNA strand and methylated at lysine-9, this kind of modification was often found at promoter regions of silenced genes

Histones are basic proteins found in chromatin and prokaryotic cells of eukaryotic organisms, which together with DNA form the nucleosome structure [8]. They are the main protein components of chromatin, act as spools for DNA fusion, and play a role in gene regulation [74]. Histones can be modified by different enzymes, and these enzymes are responsible for different functions, making epigenetic regulation very complex (Fig. 5) [75, 76]. Researchers have identified hundreds of post-translational modifications at the amino terminus or tail of histones, including phosphorylation, methylation, acetylation, ubiquitination, and carbonylation. These modifications may potentially change the structure of chromatin, leading to some gene expression and some gene silencing [11]. All core histones have metamorphic regulation regions, which are closely related to the tissue and cell environment as well as their external environment [11]. Histone modifications are also involved in the epigenetic regulation of adiposity and may play an important role in the development and progression of obesity. For example, the preadipocyte factor-1 gene (*Pref-1*) has lower levels of active chromatin markers and significantly higher H3K27 trimethylation in mesenchymal stem cells compared to committed preadipocytes. The CCAAT-enhancer-binding protein beta gene (*C/EBP*) is enriched in active chromatin markers. The *C/EBPα* is mainly marked by H3K27 trimethylation in adipogenic precursor cells, and this inhibitory marker was dramatically reduced after induction. Both *PPARγ2* and *aP2* showed to increase H3 and H4 tails acetylation during adipogenesis. Further functional studies revealed that decreased levels of H3 K27 trimethylation resulted in derepression of *Pref-1*, while increased levels of histone acetylation activated transcription of *PPARγ2* and *aP2*. Furthermore, the active histone modification-t marked 3'-UTR of *C/EBPβ* was shown to be a strong enhancer element by the luciferase assay. These results suggest that histone modifications are gene-specific in genes regulated by adipogenesis, and they play distinct roles on the regulation of the transcriptional network during adipogenesis [77]. Leung et al. [78] showed that environmental factors may promote the course of metabolic disease through chromatin alterations. They found that a high-fat diet resulted in chromatin remodeling, and led to changes in gene expression of C57BL/6 J mice livers. Moreover, the regions with largest chromatin changes were closely associated with liver transcription factors, hepatocyte nuclear factor 4-alpha (*HNF4α*), CCAAT/enhancer-binding protein alpha (*CEBP/α*), and forkhead box A1 (*FOXA1*). Further studies of DBA/2J mice revealed that the regions with the greatest chromatin changes were strain-specific and that the integration of chromatin, gene expression, and genetic data could be used to characterize regulatory regions [78]. These findings demonstrate that changes in epigenome and chromatin remodeling are strain-specific dynamics. In another study, Leung et al. [79] investigated chromatin changes in C57BL/6J and DBA/2J mice after diet reversal. Most chromatin changes in C57BL/6J mice were found to remain a remodeling state, while in DBA/2J mice most chromatin changes were transient [79]. These data suggest that the duration of chromatin changes is related to post-translational modifications of transcription factors and histones. The identified persistent loci may contribute to the overall phenotype and could be potential therapeutic targets. Funato et al. [80] determined the role of orexin in metabolic effects, which is primarily mediated by orexin receptor 2 (*OX2R*). Transgenic orexin overexpression counteracted obesity induced by the high-fat diet and insulin insensitivity by promoting energy expenditure and reducing exertion. Selective OX2R agonists inhibited diet-induced obesity, and leptin overexpression enhanced this effect, but obese mice deficient in leptin- completely lost the inhibitory effect, suggesting that leptin-OX2R signaling can exert a diet-induced MetS by improving leptin sensitivity and energy homeostasis.

Hauke et al. [81] found that histones could be modified by 4-hydroxynonenal (4-HNE). Histone carbonylation by 4-HNE was increased with age in male flies and visceral fat depots of mice, and it was also enhanced in genetic (ob/ob) mice and high-fat feeding models of obesity. In vitro proteomic evaluation revealed Michael and Schiff base adducts, but only Michael adducts were found in obese mice. Furthermore, 11 loci of 4-hydroxy hexenal (4-HHE) and 10 loci of 4-HNE histone modification were identified in visceral adipose tissue [81]. These findings suggest that histone carbonylation modifications in adipose tissue are a redox-related epigenomic marker. Currently, the prevalence of obesity and its associated complications and comorbidities is increasing globally every year, even though various interventions have been taken by all parties, but targeted approaches are still lacking. Epigenetic modifications are emerging as a bright new star that promises to be a new therapeutic tool for the development of obesity and related disciplines. Post-translational modifications of DNAm and histone synergistically build complex epigenetic signals and alter transcriptional networks in patients with cardiometabolic disorders [82]. In a previous study, Wang et al. [83] explored the effects of genome-wide histone modifications on genetic and biological processes during a high-fat diet and elucidated the relevance of gene modifications to energy metabolism and inflammation under different conditions. ChIP-seq and transcriptome in mouse adipose tissue revealed that numerous genes involved in adipogenesis, energy metabolism and inflammatory pathways were modified by H3K9me2, H3K9me3, H3K4me1 and H3K27ac histones during a high-fat diet. Meanwhile, a large number of RNA-seq and mononuclear RNA-seq datasets identified several genes in the activated mitogen-activated protein kinase (MAPK) pathway that may be involved in inflammation and energy metabolism among various genera. Immunoblot analysis also further confirmed increased expression of MET proto-oncogene, receptor tyrosine kinase (*MET*), vascular endothelial growth factor A (*VEGFA*), and increased phosphorylation of p44/42 MAPK after high-fat diet treatment [83]. This study deepens our understanding of dietary behavior in the development of obesity and may serve as a potential preventive and therapeutic target in the future. Lunke et al. [84] showed that histone deacetylation induced by valproic acid (VPA) stimulation was a major feature after vorinostat/zorinza exposure, thus defined an acetylation/deacetylation (Ac/Dc) axis. Insulin resistance refers to the state in which the amount of insulin is insufficient or does not work. Multiple factors including epigenetic modifications, can contribute to insulin resistance, and epigenetic modifications are one of the main mechanisms of obesity development. Patients with insulin resistance have lower levels of histone modifications in adipose tissues. Furthermore, many genes, such as *PPARG*, *SLC2A4* and adiponectin, C1Q and *ADIPOQ* were marked differently by histone methylation and acetylation. Among them, histone modifications of the *PPARG* and *SLC2A4* were the main genes of insulin resistance. Epigenetic changes appeared 72 h after insulin resistance induction, and were mediated through the Sirtuin (*SIRT*) family, which demonstrated the role of the *SIRT* family in insulin resistance [85]. Obesity can cause other reproductive problems such as infertility and miscarriage, and the reasons for this are related to the effects of obesity on oocytes and embryos. Pan et al. [86] used single-cell RNA transcriptome sequencing and found that 4-cell and morula/blastocyst rates were significantly lower in obese mice during embryonic development. Genome-wide analysis revealed the expression of over 1100 genes related to p53 signaling and apoptosis in 2-cell embryos. Further analysis revealed the expression changes in 47 genes associated with DNA damage, such as RAD51 recombinase (*RAD51*) and testis expressed 15, meiosis and synapsis associated (*TEX15*). In addition, obesity affected histone methylation and decreased H3K4-me2 levels. These findings suggest that obesity affects early embryonic development by inducing DNA damage, histone methylation, and autophagy in mice.

## miRNA-mediated regulation

**Fig. 6:**
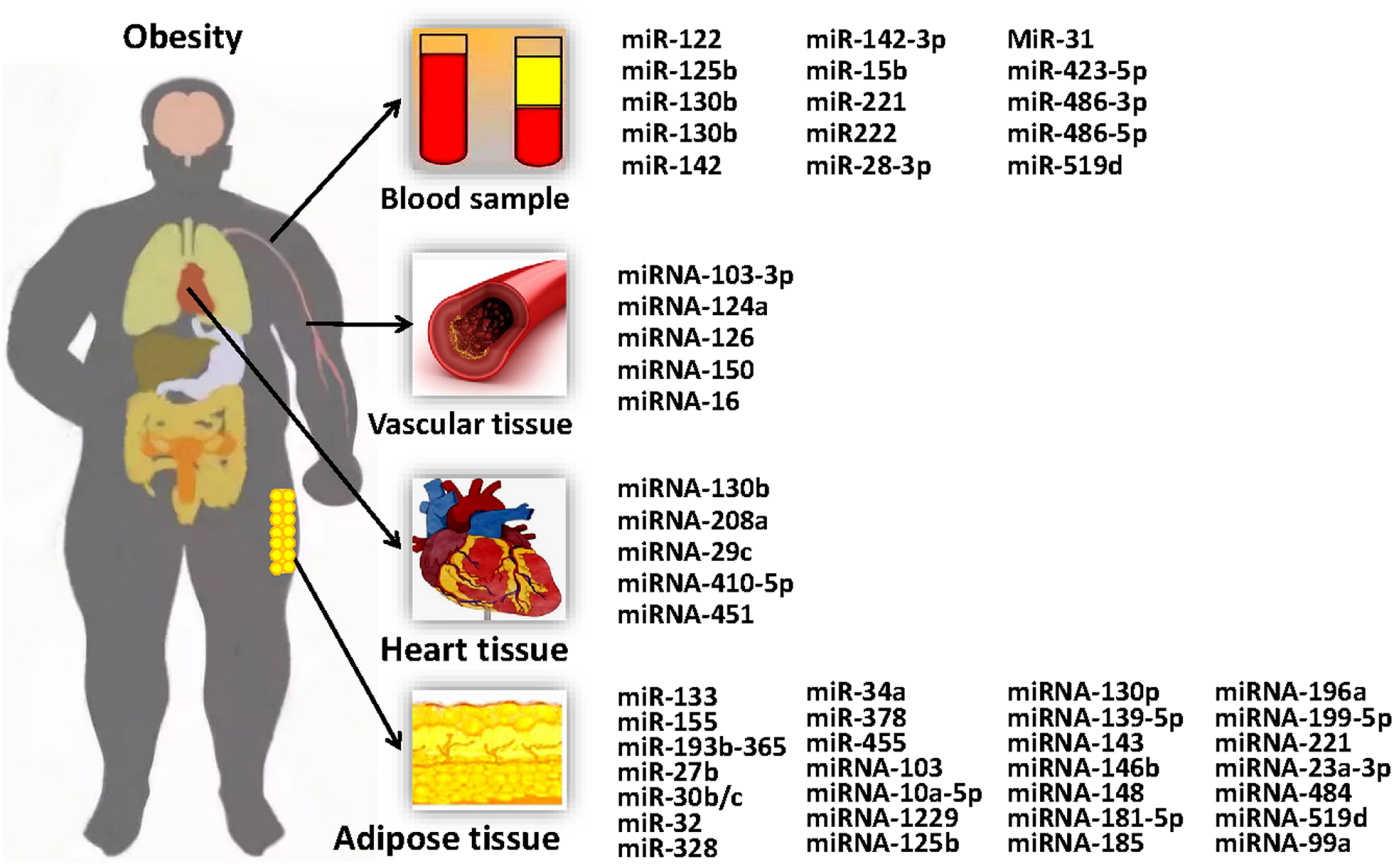
Dysregulation of obesity-associated miRNAs in different biological specimens. It includes blood sample and vascular, heart, and adipose tissues

MicroRNAs (miRNAs) are a very important part of non-coding RNAs (ncRNAs) and are closely associated with key regulators of metabolic abnormalities [87]. miRNAs are short noncoding RNA sequences of 18 to 25 nucleotides in length that can regulate gene expression and silence [88]. miRNAs has been present in all eukaryotic cells conserved in the species since they were discovered in 1993. miRNAs regulate gene expression by inducing cleavage of mRNAs or inhibiting protein translation, and binding to complementary sequences [89]. miRNAs play key role in metabolic processes, and their dysregulated expression has been reported in obesity (Fig. 6) [90, 91]. Increasing evidence supports the role of miRNAs as key factors between adipose tissue, liver, skeletal muscle, and other organs that allow paracrine communication between different tissues [87]. The regulatory role of miRNAs in adipose tissue (AT) can influence metabolism and energy homeostasis, especially in mammals where white adipocytes, brown adipocytes, and induced brown adipocytes in white adipocytes are present. In fact, several miRNAs have been found to regulate key signaling pathways for adipogenesis in brown adipocytes, brite AT, and white adipocytes by promoting or repressing transcription factors for adipocyte differentiation. For example, miR-328, miR-378, miR-30b/c, miR-455, miR-32 and miR-193b-365 activate brown adipogenesis, while miR-34a, miR-133, miR-155 and miR-27b inhibit brown adipogenesis. It is well known that white adipocytes store energy mainly in the form of lipids, whereas and brown adipocytes dissipate energy mainly in the form of calories. The role of miRNAs in different types of AT has attracted the interest of many researchers who hope that miRNAs can be targeted to find treatments for obesity, diabetes and other related diseases [92]. Scientists have identified more than 2500 mature miRNAs in the human genome [93]. While more than 60% of genes in the human genome are regulated by miRNAs [94], one miRNA can target and regulate thousands of different mRNAs, and one mRNA can also be regulated by multiple miRNAs [95]. Thus, miRNAs are considered to be regulators of key genes in various biological processes that can be regulated, including adipocyte proliferation, insulin resistance differentiation, and inflammation [96]. Remarkably, AT is a major source of circulating miRNAs, which have recently been described as novel adipokines. Several AT-derived miRNAs are closely associated with adipocyte differentiation, obesity-related insulin resistance and inflammation, and changes in the tumor microenvironment. During obesity, AT can completely alter the profile of secreted miRNAs, affect circulating miRNAs, and influence the development of different pathological conditions, such as obesity, MetS, and cancer [97]. There is evidence of associations between studies that the genetic predisposition to obesity in humans is associated with specific high levels of miRNAs [98]. Prats-Puig et al. [99] suggested that circulating miRNAs are valuable as a biomarker for potential therapeutic targets in metabolic diseases. They define the circulating patterns of miRNAs in childhood obesity. This cross-sectional validation study identified 15 circulating miRNAs that were significantly dysregulated in prepubertal obesity. Among them, miR-28-3p and miR-221 were down-regulated, while miR-423-5p, miR-130b, miR-142-3p, miR-486-3p, and miR-486-5p were up-regulated. Circulating concentrations of these miRNAs were correlated with BMI and other indicators of obesity. The 3-year follow-up revealed changes in circulating miRNA levels that accompany weight changes in children [99], suggesting the possibility of performing an early detection of circulating miRNA and using it to monitor children for the presence of metabolic abnormalities. In addition, circulating miRNA levels were also associated with insulin resistance [100], early childhood obesity [101], MetS in obese adolescents [102], neonates whose mothers were overweight and obese before pregnancy [103], obesity-associated inflammatory and metabolic diseases in pediatric patients [104]. Zhao et al. [105] identified 23 important plasma miRNAs that can be used as biological markers of weight gain. MiR-31, miR-519d, miR222, miR-130b, miR-15b, miR-125b, miR-122 and miR-142 of individuals had a susceptibility to obesity and more than three times higher odds of weight gain than the general population. These findings support the rationale that circulating miRNAs play an important role on obesity and weight gain. Exosomal miRNAs, an emerging subject of study in recent years, have been found to correlate their levels in adipocytes with weight loss and improved insulin resistance after gastric bypass surgery in obese patients [106]. All these evidences support the key role of miRNAs on obesity and related metabolic disorders, which could serve as biomarkers or potential therapeutic targets. To determine the impact of adipogenic miRNAs on the adipocyte differentiation process, Shi et al. [107] used microarray technology to monitor miRNA levels in differentiated adipocytes, human stromal vascular cells, and human adipose-derived mesenchymal stem cells. A total of 79 differentially expressed miRNAs were identified, most of which were located in chromosomal regions associated with obesity but not associated with adipocyte differentiation processes. A systematic search was also performed for the relevant studies in several academic databases, including GEO Array Express, Pubmed and Embase, and finally 8 studies on human adipocyte differentiation and obesity were included. The meta-analysis identified 42 differentially expressed miRNAs that were specific for adipogenesis, several of which were associated with key gene targets, mainly enriched in pathways such as lipid metabolic, cell cycle, cell differentiation, MAPK signaling, insulin receptor signaling pathway and Wnt signaling process. These results suggest that circulating miRNAs play a great role in adipogenesis and cellular differentiation processes, which could provide new potential therapeutic directions for obesity and related metabolic diseases.

Changes in miRNA expression were also observed in tissues, but the patterns were quite different between tissues. Differentially expressed miRNAs and their targeting pathways have also been found in populations with obesity interventions through dietary or surgical approaches. This has led to a deeper understanding of obesity and related metabolic diseases [108]. Oxidative stress (OxS), a cause and consequence of MetS, has the ability to trigger the dysregulation of metabolic and epigenetically related signaling pathways, including various miRNAs. Studies have shown that OxS from different sources alters the expression of miRNAs in various tissues and cells. Dysregulated miRNAs directly or indirectly affect the expression of antioxidant signaling pathways and effector enzymes, ROS producers (e.g. NOX4/5), and genes of numerous signaling pathways related to inflammation, insulin sensitivity, and lipid metabolism. miRNAs also appear to be important epigenetic modifiers that regulate redox responses to achieve the biological effects of regulatory homeostasis, suggesting that miRNAs may be a promising direction to improve OxS status in MetS [109]. Intact endothelial cells maintain normal physiological function of the vasculature, but obesity disrupts its integrity and dysfunction, thus becoming a high risk factor for cardiovascular disease, including endothelial nitric oxide synthase (eNOS), Sirtuin 1 (SIRT1), oxidative stress, autophagic mechanisms, and endoplasmic reticulum stress are involved in this process. All of these factors are directly or indirectly related to miRNAs, and therefore therapeutic modalities targeting miRNAs may have positive therapeutic implications for obesity and related cardiovascular diseases [110].

## Effects of intrauterine and early development environment

Obesity and its related metabolic disorders have become a very common disease among the general populations. In the past, the poor lifestyle and improper diet were considered the main causes of its development. In recent years, however, there have been new findings that, in addition to lifestyle and eating habits after birth, obesity and its related metabolic disorders may have roots in humans before birth. Some of the better known theories, including the “fetal origin” theory or the “fetal programming” theory, are based on the conclusion that environmental factors alter the development of the uterus in the mother, leading to the later development of cardiovascular disease and MetS. The reason for this is not based on genetic defects, but on changes in gene expression as the fetus adapts to its environment during development. The endocrine system is influenced by fetal development, but recent studies have identified leptin and insulin resistance as key endocrine changes in obesity and related metabolic disorders [111]. Many studies have elucidated the relationship between changes in the early life environment and susceptibility to obesity and related metabolic disorders. Pregnant women who are malnourished or obese promote the intergenerational transmission of metabolic disorders (Fig. 1). This has also been termed the developmental programming hypothesis, whereby changes in environmental factors during critical developmental periods can have permanent effects on offspring. However, current evidence suggests that this developmental programming is a transgenerational phenomenon and that even in the absence of environmental factors, this developmental programming effect is transmitted to offspring, triggering metabolic disorders and obesity. The mechanisms underlying the hypothesis of developmental programming are still unclear, but there is evidence that epigenetic modifications facilitate the transformation of this process. However, it has also been suggested that interactions between non-genomic components and the fetus and the environment with the uterus also play an important role [112]. The Developmental Origins of Health and Disease hypothesis highlights the link between prenatal, perinatal, and early postnatal exposure to certain environmental factors and obesity and non-communicable diseases. Fetal overnutrition caused by maternal obesity and overweight is a major cause of obesity and metabolic disorders in offspring. Pregnancy is a special time when various physiological functions and hormone levels change, and some lifestyle changes in the mother during this period may have health benefits for the child [113]. Using the paradigm of fetal programming, Vickers et al. [114] established an animal model to study the common phenotypes of obesity and metabolic disorders in humans. They also created a nutrient-deficient intrauterine environment using maternal malnutrition throughout pregnancy to induce fetal programming. Maternal malnutrition can lead to retardation of fetal growth and significant weight loss at birth. Programmed offspring frequently developed hyperphagia, hyperinsulinemia, obesity, and other diseases in adulthood. In recent years, one of the most important discoveries has been the relationship between intrauterine experience and later metabolic and cardiovascular disease.

Changes in the fetal environment have been shown to have a profound impact on endocrine and metabolic systems in adulthood. These phenomena are called “fetal programming”. Maternal malnutrition during the pregnancy period could lead to obesity, hyperinsulinemia, high blood pressure, hyperleptin, and hyperphagocytosis of the offspring, in later life [115, 116]. Stein et al. [117] investigated the effect of famine on maternal birth weight (MBW) and the relationship between MBW and the birth weight of offspring. Findings from a survey of women who had given birth before the Dutch famine of 1944–1945 found that the prenatal famine affected MBW and did not affect the mean birth weight of mothers and offspring. Three months of exposure were not a determinant of the birth weight of the offspring, but acute famine may indirectly affect the birth weight of offspring through its alteration of MBW. Veenendaal et al. [118] found that when women had malnutrition during pregnancy, their grandchildren had a higher probability of neonatal obesity. Their further study showed that offspring from prenatally malnourished fathers (not mothers) were more likely to be overweight and obese than offspring whose parents were both prenatally malnourished, and increased obesity of former offspring may increase the incidence of various chronic diseases. Recently, scholars have identified the effects of developmental programming on children as a major cause of the obesity epidemic, particularly in infants who born to mothers with obesity or diabetes, newborns who have experienced fetal growth restriction, and offspring who have been exposed to environmental toxins. The main mechanisms that lead to obesity in offspring include hypothalamic appetite programming pathways and adipogenic pathways. Processes include changes in the precursor cells of appetite/satiety neuron and the adipose cells, epigenetic modifications, and nutrient sensors that can be modulated to improve adiposity in offspring [119]. The interactions of genes and nutrients on energy homoeostasis and metabolism suggest that inflammation may not only a comorbidity but also a cause of obesity. Some instances of the action of nutritional interventions including excess fat intake, protein restrictions, and energy deprivation during pregnancy and lactation showed that a variety of diseases and different metabolic pathways influence the energy efficiency of offspring. These diseases are regulated by epigenetics. Maternal dietary habits, environmental risk factors, and intergenerational or transient transmission can facilitate or inhibit epigenetic mechanisms, which may be related to the susceptibility of offspring to inflammation and obesity. Researchers are currently conducting various basic and clinical trials to identify relevant epigenetic biomarkers at an early stage of disease onset and to specify appropriate prevention and treatment recommendations to prevent disease onset or progression. These approaches will help to achieve personalized “epigenomic models” of therapeutic strategies to combat obesity and the various complications associated with it [120]. At this moment, the various complications associated with obesity are increasing with the rising prevalence of obesity. In addition, studying the changes that occur in the maternal environment during pregnancy can also help to understand the pathogenesis and genetic mechanisms between generations of obesity and metabolic dysfunction [121].

The intrauterine environment plays a key role in the development of the fetus and in the long-term epigenetic changes that the offspring may have. Painter et al. [122] paid a visit to cohort members (F1) who were born during the famine in the Netherlands in 1944 to 1945 and were exposed (or not) to the famine in utero, and their offspring (F2). No transgenerational effects of prenatal famine on birth weight and prevalence of cardiovascular and metabolic diseases were observed. However, the generation of F1 after exposure to intrauterine famine status was associated with an increased probability of obesity and unhealthy conditions in F2 newborns, suggesting that the effects of intrauterine famine exposure are not only limited to the generation of F1, but have a persistent effect on the generation of F2. Stanner et al. [123] also showed that intrauterine malnutrition in adults was not related to impaired glucose tolerance, blood lipid disorders, high blood pressure, or other diseases. Endothelial dysfunction has been observed in individuals exposed to malnutrition, but obesity has a broader effect on blood pressure. The intrauterine group had elevated concentrations of von Willebrand factor compared to the infant group, and the interaction between obesity and blood pressure values was strongly associated in female subjects in the intrauterine group. Short stature in adults was associated with increased insulin and blood glucose concentrations two hours after glucose loading. Compared to the exposed group, subjects in the unexposed group showed non-systematic differences in coagulation factors, diastolic blood pressure, and subscapularis-triceps skinfold ratio. Sparén et al. [124] studied the determined long-term health effects of adulthood. Cardiovascular risk factors and mortality were analyzed from 1975 to the end of 1999. The results showed that starvation or concomitant chronic stress, may increase susceptibility to cardiovascular disease, especially during adolescence. Using fetal undernutrition throughout pregnancy, Vickers et al. [125] investigated whether fetal programming mediates the development of obesity and hypertension in adulthood through insulin and leptin, and further investigated whether increased appetite is a trigger for disease. Unproduced Wistar rats were randomly assigned to the ad libitum group (AD group), the 30% ad libitum group, and the undernourished group (UN group) after mating. The offspring rats were then assigned to the 30% high-fat diet group and the control diet group after weaning. It was found that the food intake of the offspring in the UN group increased significantly in the early postnatal period and with age, and the effect was more pronounced in the high-calorie diet. In addition, a significant increase in systolic blood pressure, fasting glucose and insulin levels was observed in the offspring of the UN group [125]. This study suggests that adult bulimia is a result of fetal programming and that hyperinsulinemia and hyperleptinemia may play a key role in the development of diseases such as hyperphagia, obesity, and hypertension. Epigenetic mechanisms are also increasingly being suggested to mediate environmental exposures in early pregnancy with programmed changes in gene expression associated with offspring growth and development. Both basic studies from animals and clinical studies suggest that exposure to environmental factors during pregnancy can influence child development and the development of obesity and MetS related to obesity. However, this effect of transmission to offspring occurs in the absence of sustained exposure to environmental factors, which in turn results in the continued spread of the cycle of obesity and MetS. This phenomenon may be due to extrinsic processes. In addition, epigenetic inheritance can be transmitted through somatic or germ cells. In conclusion, epigenetic modifications are currently considered partially reversible, and understanding the mechanisms that contribute to obesity and metabolic dysfunction has important implications for early identification and intervention in disease [126].

## Effects of endocrine disrupting chemicals

Endocrine disruptors (EDs) or endocrine disrupting chemicals (EDCs) are defined as environmental pollutants (chemicals) that can interfere with the functioning of the hormonal system. They are continuously released into the environment as food additives, electronic waste, and synthetic fertilizers, and can be considered obesogenic compounds (obesogens) because they can alter adipogenesis and lipid metabolism processes [127]. The number and types of EDCs are increasing year by year, and some of these stable substances persist in the environment without degradation [128]. EDCs include smoke-derived compounds, polybrominated diphenyl ether, “non-persistent” phenolic compounds, heavy metals, and persistent organic pollutants [127]; flame retardants, detergents, plastics, industrial and household products, and pesticides/herbicides [129]; phthalates, perfluoroalkyl substances [130]; ethylbenzene, m/p-xylene, o-xylene, toluene, cyclohexanone, 2-butoxyethanol, butylated hydroxytoluene, cyclohexanone, styrene, and hexane [131]; monomers of plastic materials, polycarbonates, epoxy resins [132]; triclosan [127, 130] and Bisphenol A (BPA) [130, 132]. Epidemiological and experimental studies have shown that the fetal or neonatal period in utero is a particularly sensitive time for exposure. The lipophilic structure allows the EDCs to increase their own retention capacity during fat deposition. This may create a vicious cycle that induces obesity while increasing the retention of contaminants with broader adverse effects. This is one of the explanations for obesity as a potential risk factor for many diseases [129]. EDCs may increase the risk of diseases in children by disrupting hormones that play a vital role in growth and development. Rapid development and increased exposure to certain EDCs in fetuses, infants, and children can increase sensitivity to environmental stressors (e.g. EDCs) due to developmentally specific behaviors, anatomy, and physiology. Ultimately, improving the potential health risks of exposure of EDCs to children’s health can help identify vulnerable groups and develop appropriate public health interventions to reduce exposure [130]. Indoor air pollutants may act as EDCs. However, the extent of health damage caused by contaminants remains unclear. Paciência et al. [131] assessed the association between exposure to EDCs and obesity in schoolchildren. Scientists found that classrooms with more obese children also had higher overall levels of EDCs. Higher levels of cyclohexanone were associated with increased BMI, and higher levels of 2-butoxyethanol, butylated hydroxytoluene, cyclohexanone, styrene, and hexane were associated with obesity. These findings further support the role of EDCs in promoting the development of obesity. Furthermore, even low levels of EDCs increase the risk of obesity. Heras-González et al. [132] estimated dietary exposure to phytoestrogens and BPA and their potential obesogenic effects in a group of Spanish schoolchildren. The diet of this group had an average total estrogenic volume of 5.10–12 M eq.E2 (5 pmol/day). In this pediatric population, the study found that being normal weight was significantly associated with the probability that obesity organism activity consumed energy and the total proliferative effect, with less active children at high risk of obesity.

Certain people were susceptible to EDCs due to environmental conditions and unhealthy diet. Obesity caused by exposure to EDCs was an evolving reality [133]. Yan et al. [134] showed that maternal rats exposed to polycyclic aromatic hydrocarbons (PAHs) during gestation had offspring that were more prone to overweight and increased adiposity, along with increased gene expression of adiponectin, fatty acid synthase (*FAS*), cyclooxygenase-2 (*Cox-2*), *C/EBPα*, CCAAT/enhancer-binding proteins alpha, and *PPARG*, but decreased DNAm of *PPARG*. Similar differences in phenotype and DNAm extended to grandchild mice. These results suggest that prenatal PAH exposure is associated with changes in body weight, adiposity, adipose gene expression, and epigenetics in offspring. Among these epigenetic changes, there are important mediators linking environmental damage during development with susceptibility to disease in adulthood. Animal studies are focusing on the direct relationship between perinatal BPA exposure and DNAm, but these studies are often limited in terms of candidate genes. Anderson et al. [135] studied epigenetic changes in liver tissue in mice after 10 months of perinatal BPA exposure at physiological doses through agenomic platform of the whole epigenome. Finally, metabolic pathways enriched in differentially methylated regions were analyzed and four candidate genes (transmembrane protein 238, *Tmem238*; regulatory factor X-related protein, *Rfxap*; retinoid X receptor, *Rxr*; and Janus kinase 2, *Jak-2*) were selected to evaluate the role of DNAm as mediator. DNAm in all four candidate genes was found to be associated with developmental BPA exposure and energy expenditure, body weight, and body fat, which are critical to understanding epigenetics in the pathogenesis of chronic diseases and for developing new treatments and drugs. These findings also suggest that animal and clinical studies have ample evidence of epigenetic mechanisms in EDCs on childhood obesity and metabolic dysfunction. Smith et al. [136] argued that determining obesity by BMI in the clinical evaluation of the health effects of EDCs is itself a confounding factor. Studies have shown that EDCs have the highest risk in the early stages of fetal and infant development, when the development of different organs and systems is taking shape. Exposure to EDCs in the general non-specific selected population is very low, and exposure measurements are lacking, making epidemiological studies of EDCs in the general population difficult to conduct. If obese patients are to be included in epidemiological studies of hormone-related effects, subjects should ideally be classified by body fat percentage rather than BMI.

## Conclusion and future outlook

The process of altering gene activity without modifying the nucleotide sequence is defined as epigenetics. It controls gene activity and the growth and development of the body. Epigenetic changes are often reversible because genes can be turned on or off by “switches” through environmental intervention. Therefore, it has attracted the wide attention of the academic community and the public. The most relevant epigenetic mechanisms involved in the regulation of gene activity include DNAm, histone modifications, and miRNA-mediated processes, and the disruption of these balances can lead to the development of complex diseases such as obesity and its associated metabolic disorders. Although epigenetics is an emerging discipline, significant progress has been made in the field of study on the association between epigenetics and obesity. DNAm is the most investigated epigenetic mechanism, either at the global, loci-specific, or genome-wide levels. Differential expression of DNAm is generally considered to be the result of epigenetic dysregulation, which is linked to several phenotypes, pathologies, and adverse environments. Preliminary evidence supports the effect of epigenetics on obesity. These studies reported epigenetic changes in key metabolically important tissues following a high-fat diet, as well as epigenetic differences between lean and obese animals. Advances in DNAm research and the obesity research model may identify new DNAm markers of obesity and its related complications. The first epigenetic markers of obesity detectable at birth have been identified, which can help predict obesity risk, obesity, and body size, and inform treatment and prevention strategies. Studies have also reported the differential expression status of multiple genes before and after obesity interventions and have identified multiple candidate genes and biological markers. Therefore, DNAm markers could improve the success of weight loss treatment in the context of precision nutrition. There are currently human EWAS and numerous studies exploring the relationship between the environment, epigenome, and complex disease states that have identified epigenetic changes associated with nutrition, weight loss, and exercise. Adult susceptibility to obesity has an early developmental origin and follows an intergenerational cycle. There is also evidence that environmental exposure, including exposure to malnutrition, is associated with methylation changes and therefore has the potential influence on adult phenotypes, suggesting that transient environmental effects experienced early in life may lead to permanent effects in the form of increased disease risk in later life [137]. Furthermore, environmental exposure during key developmental periods can affect the distribution of epigenetic markers and contribute to obesity. Ultimately, this can help predict an individual’s risk of obesity at a young age and opens possibilities for introducing targeted prevention and treatment strategies. In particular, some epigenetic markers can be modified through altered exposure in utero, as well as lifestyle changes in adult life, implying the potential to introduce interventions in postpartum life to alter unfavorable epigenomic profiles. The effects of epigenetics on obesity and metabolic disease have increased rapidly, with increasing evidence linking epigenetic modifications to metabolic health outcomes. Recent studies have also emerged as potential epigenetic biomarkers. Validation of epigenetic markers in multiple cohorts, discovery of several markers in genes associated with obesity development, and combination of overlapping epigenetic markers with known obesity loci reinforce evidence that these associations are real. With the rise of higher coverage-based sequencing methods becoming more available and cost-effective, more discoveries are likely to be made, and epigenetics can improve the understanding of the complex etiology of obesity and help us better understand the complex diseases. The current findings have the potential to develop new strategies for early prevention and treatment. These findings provide support for the underlying epigenetic programming of obesity and comorbidities. On the contrary, the association between histone modifications and obesity in humans is less studied, but some of the current results suggest an association between genome-wide histone modifications and obesity. Taken together, all these studies confirm that epigenetics can be used to assess metabolic risk and personalize the clinical treatment of obesity. However, a large part of the human epigenome remains a black box waiting to be discovered. Furthermore, epigenetic studies face more challenges than genetic association studies, the most important being that the epigenome is cell- or tissue-specific, and changes over time. Only by fully understanding the underlying genetic and epigenetic mechanisms, and the metabolic processes they control, can we manage and ultimately prevent obesity. Therefore, it is necessary to conduct further research in humans to analyze the effects of nutritional status, dietary patterns, lifestyle factors, physical activity intensity, environmental pollution, and psychological factors on human health through epigenetic mechanisms.

## Data Availability

Not applicable.

## Abbreviations

*ABCA17P*: ATP-binding cassette, sub-family A (ABC1), member 17, pseudogene
*ABCG1*: ATP binding cassette subfamily G member 1
*ACSF3*: Acyl-CoA synthetase family member 3
*ADCY3*: Adenylate cyclase 3
*ADCYAP1R1*: Adenylate cyclase activating polypeptide receptor 1
*ADPN*: Adiponectin
*ADRB3*: Adrenoceptor beta 3
ALSPAC: Avon Longitudinal Study of Parents and Children
*ANGPT4*: Angiopoietin 4
*APOA5*: Apolipoprotein A5
ARIC: Atherosclerosis Risk in Communities
*ASB2*: Ankyrin repeat and SOCS box containing 2
AT: Adipose tissue
*ATOH8*: Atonal bHLH transcription factor 8
*ATP10A*: ATPase phospholipid transporting 10A
*ATP2C2*: ATPase secretory pathway Ca^2+^ transporting 2
*BAI3* (*ADGRB3*): ABI family member 3 (Adhesion G protein-coupled receptor B3)
*BBS2*: Bardet-Biedl Syndrome 2
*BCAS3*: BCAS3 microtubule associated cell migration factor
*BCAT1*: Branched chain amino acid transaminase 1
*BCL2*: BCL2 apoptosis regulator
*BCL2L2*: BCL2 like 2
*BCL9L*: B-Cell Lymphoma 9-like protein
BMI: Body mass index
BMIz: Body mass index z-scores
*BMPR1B*: Bone morphogenetic protein receptor type 1B
BPA: Bisphenol A
*BRDT*: Bromodomain testis associated
*C3orf70*: Chromosome 3 open reading frame 70
*CACNA1C*: Calcium voltage-gated channel subunit alpha1 C
*CCNL1*: Cyclin L1
*CD36*: Cluster Determinant 36
*CD38*: Cluster of differentiation 38
*CDC3*: Cell division cycle 37
*CDKAL1*: Cyclin-dependent kinase 5 regulatory subunit associated protein 1 like 1
*CES1*: Carboxylesterase 1
*CFLAR*: CASP8 and FADD like apoptosis regulator
*CHN2*: Chimerin 2
*CILP2*: Cartilage intermediate layer protein 2
*CISH*: Cytokine inducible SH2 containing protein
*CLDN1*: Claudin 1
*CMIP*: C-Maf inducing protein
*COMT*: Catechol-O-methyltransferase
*COX6A1P/FGD2*: Cytochrome C oxidase subunit 6A1 pseudogene /FYVE, RhoGEF and PH domain containing 2
*CPSF4L*: Cleavage and polyadenylation specific factor 4
*CPT1A*: Carnitine palmitoyltransferase 1A
*CRH*: Corticotropin releasing hormone
*CRHR2*: Corticotropin releasing hormone receptor 2
*CRISP2*: Cysteine rich secretory protein 2
*CTSZ*: Cathepsin Z
*CTTNBP2*: Cortactin binding protein 2
*CxCl10*: C-X-C motif chemokine ligand 10
*CXCL2*: C-X-C motif chemokine ligand 2
*CYTH4-ELFN2*: *C*Ytohesin 4, extracellular leucine-rich repeat and fibronectin type III domain containing 2
*DDX10*: DEAD-box helicase 10
*DHCR24*: 24-Dehydrocholesterol reductase
*DISC1*: Disrupted-in-Schizophrenia-1
*DLC1*: DLC1 Rho GTPase activating protein
*DLX5*: Distal-less homeobox 5
*DMAP1*: DNA methyltransferase 1 associated protein 1
DMC: Differentially methylated CpG loci
DMCpG: Differentially methylated CpG
DML: Differentially methylated DNA loci
DMP: Differentially methylated position
*DNAJC27*: DnaJ heat shock protein family (hsp40) member C27
DNAm: DNA methylation
*DNM2*: Dynamin 2
DNMT: DNA methyltransferase
DVC: Differentially variable CpG loci
*DYRK2*: Dual specificity tyrosine phosphorylation regulated kinase 2
ED: Endocrine disruptor
EDC: Endocrine disrupting chemical
*EGFL7*: EGF like domain multiple 7
*ETBP1*: SET binding protein 1
*ETV5*: ETS variant transcription factor 5
EWAS: Epigenome-wide association studies
*FAM3C*: FAM3 metabolism regulating signaling molecule C
*FBP2*: Fructose 1,6-bisphosphatase 2
FDR: False discovery rate*FGFRL1*: Fibroblast growth factor receptor like 1
FH: Familial hypercholesterolemia
*FHL2*: Four and a half LIM domains 2
*FMN1*: Formin 1
*FSD2*: Fibronectin type III and SPRY domain containing 2
*FSTL1*: Follistatin like 1
*FTH1P20*: Ferritin heavy chain 1 pseudogene 20
*FTO*: FTO alpha-ketoglutarate dependent dioxygenase
*FYN*: FYN oncogene related to SRC, FGR, YES
*GALE*: UDP-galactose-4-epimerase
GBS: Gastric bypass surgery
*GBX2*: Gastrulation brain homeobox 2
GENOI: Grupo Navarro de Obesidad Infantil
*GGT1*: Gamma-glutamyltransferase 1
*GRIN2D*: Glutamate receptor, ionotropic, N-methyl-D-aspartate receptor subtype 2D
*HAND2*: Heart and neural crest derivatives expressed 2
*HDAC4*: Histone deacetylase 4
*HERC2*: HECT and RLD domain containing E3 ubiquitin protein ligase 2
*HHEX*: Hematopoietically expressed homeobox
*HID1*: HID1 domain containing
*HIF3A*: Hypoxia inducible factor 3 alpha subunit
*HK1*: Hexokinase 1
*HMGA1*: High mobility group AT-hook 1
*HOXA3*: Homeobox A3
*HOXB1*: Homeobox B1
*HOXB3*: Homeobox B3
*HOXC10*: Homeobox C10
*HOXC6*: Homeobox C6
*HOXC9*: Homeobox C9
*HOXC-AS2*: HOXC Cluster Antisense RNA 2
*HOXC-AS3*: HOXC Cluster antisense RNA 3
*HOXD10*: Homeobox D10
*HOXD8*: Homeobox D8
*HOXD9*: Homeobox D9
*HRASLS2*: HRAS-like suppressor 2
*HUWE1*: HECT, UBA and WWE domain-containing 1
*ICAM-1*: Intercellular adhesion molecule 1
*IFI44L*: Interferon induced protein 44 like
*IGF2BP1*: Insulin like growth factor 2 MRNA binding protein 1
IGFBPL1: Insulin like growth factor binding protein like 1
*IGHE*/*IGHG1*/*IGHD*: Immunoglobulin heavy constant epsilon / immunoglobulin heavy constant gamma 1/ immunoglobulin heavy constant delta
*INTU*: Inturned planar cell polarity protein
*IP6K1*: Inositol hexakisphosphate kinase 1
*ISL2*: ISL lim homeobox 2
*KCNQ1*: Potassium voltage-gated channel subfamily Q member 1
*KDM2B*: Lysine demethylase 2B
*KIF15*: Kinesin family member 15
*KLF4*: Kruppel-like factor 4
*KLHL18*: Kelch-like family member 18
*KLHL6*: Kelch like family member 6
KORA: Cooperative Health Research in the Region of Augsburg
*KRT16*: Keratin 16
*LARS2*: Leucyl-tRNA synthetase 2
*LCAT*: Lecithin-cholesterol acyltransferase
LDL-C: Low-density lipoprotein cholesterol
*LEP*: Leptin
*LEPR*: Leptin receptor
*LGALS2*: Galectin 2
*LGALS3BP*: Galectin 3 binding protein
*LINE-1*: Long interspersed element-1
*LMX1A*: LIM homeobox transcription factor 1, alpha
*LRRC27*: Leucine rich repeat containing 27
*MADD*: MAP kinase activating death domain
*MAGI2*: Membrane associated guanylate kinase, WW and PDZ domain containing 2
*MAP1A*: Microtubule associated protein 1A
*MAP2K3*: Mitogen-activated protein kinase kinase 3
*MAP2K5*: Mitogen-activated protein kinase kinase 5
*MAP3K5*: Mitogen-activated protein kinase kinase kinase 5
MAPK: Mitogen-activated protein kinase
*MAST3*: Microtubule associated serine/threonine kinase 3
MBW: Maternal birth weight
*MC2R*: Melanocortin 2 receptor
*MC4R*: Melanocortin 4 receptor
*MCF2L*: MCF.2 cell line derived transforming sequence like
MetS: Metabolic syndrome
miRNA: MicroRNA
*MLLT4*: Myeloid/lymphoid or mixed-lineage leukaemia, translocated to 4
*MPG*: N-methylpurine DNA glycosylase
*MRPS33*: Mitochondrial ribosomal protein S33
*MSI2*: Musashi RNA binding protein 2
*MSRA*: Methionine sulfoxide reductase A
*MYH11*: Myosin heavy chain 11
*MYL6*: Myosin light chain 6
*MYT1L*: Myelin transcription factor 1 like
*NCAM1*: Neural cell adhesion molecule 1
*NCAPH2*: Non-SMC condensin II complex subunit H2
*NCOR2*: Nuclear receptor corepressor 2
ncRNA: Non-coding RNA
*NEGR1*: Neuronal growth regulator 1
NEST: Newborn Epigenetics Study
*NFATC2IP*: Nuclear factor of activated T cells 2 interacting protein
*NLRC5*: Nucleotide-binding oligomerization domain-like receptor family caspase recruitment domain containing 5
*NR1H3*: Nuclear receptor subfamily 1, group H, member 3
*NRF1*: Nuclear respiratory factor 1
*NRXN3*: Neurexin 3
*NUDT3*: Nudix hydrolase 3
*NXPH1*: Neurexophilin 1
OxS: Oxidative stress
*P2RX4*: Purinergic receptor P2X 4
PAH: Polycyclic aromatic hydrocarbon
*PBX1*: PBX homeobox 1
*PCK2*: Phosphoenolpyruvate carboxykinase 2, mitochondrial
*PER3*: Period3
*PHACTR1*: Phosphatase and actin regulator 1
*PHGDH*: Phosphoglycerate dehydrogenase
*PIM3*: Pim-3 proto-oncogene, serine/threonine kinase
*PITX2*: Paired like homeodomain 2
*PIWIL4*: Piwi-like RNA-mediated gene silencing 4
*PLCH1*: Phospholipase C eta 1
*PNKD*: PNKD metallo-beta-lactamase domain containing
*PNOC*: Prepronociceptin
*POMC*: Proopiomelanocortin
*PPARG*: Peroxisome proliferator activated receptor gamma
*PPARGC1B*: PPARG coactivator 1 beta
*PRDM14*: PR/SET domain 14, previously PR domain containing 14
*PRDM16*: PR/SET Domain 16
*PRDM2*: PR/SET domain 2
*PROC*: Protein C, Inactivator of coagulation factors Va and VIIIa
*PROX1*: Prospero homeobox 1
*PSMD1*: Proteasome 26S subunit, non-atpase 1
*PTPRS*: Protein tyrosine phosphatase receptor type S
*PXDN*: Peroxidasin
*RADIL*: Rap associating with DIL domain
*RALBP1*: RALA binding protein 1
*RAX2*: Regulator of axillary meristems 2
RODAM: Research on Obesity and Diabetes among African Migrants
*RORC*: RAR related orphan receptor C
*RPS6KA2*: Ribosomal protein S6 kinase A2
*RPTOR*: Regulatory associated protein of MTOR, complex 1
*RUNX2*: RUNX family transcription factor 2
*SBNO2*: Strawberry notch homolog 2
*SCD1*: Stearoyl-CoA desaturase 1
*SFRP2*: Secreted frizzled related protein 2
*SFRP5*: Secreted frizzled-relate protein 5
*SFXN5*: Sideroflexin 5
*SIM1*: Single-minded homolog 1
*SKAP2*: Src kinase associated phosphoprotein 2
*SLC22A11*: Solute carrier family 22 member 11
*SLC22A8*: Solute carrier family 22 member 8
*SLC25A20*: Solute carrier family 25, member 20
*SLC35F3*: Solute carrier family 35 member F3
*SLC39A12*: Solute carrier family 39 member 12
*SLC43A2*: *S*Olute carrier family 43 member 2
*SLC5A11*: Solute carrier family 5 member 11
*SLFN12*: Schlafen family member 12
*SMAD3*: SMAD family member 3
*SNED1*(*IRE-BP1*): Sushi, nidogen and EGF like domains (insulin-responsive sequence DNA-binding protein 1)
*SOCS3*: Suppressor of cytokine signaling 3
*SORBS2*: Sorbin and SH3 domain containing 2
*SORBS3*: Sorbin and SH3 domain containing 3
*SORCS2*: Sortilin related VPS10 domain containing receptor 2
*SOS1*: SOS Ras/Rac guanine nucleotide exchange factor 1
*SOX2OT*: SOX2 overlapping transcript
*SREBF1*: Sterol regulatory element binding transcription factor 1
*ST3GAL4*: ST3 beta-galactoside alpha-2,3-sialyltransferase 4; *ST6GAL1*: ST6 beta-galactosamide alpha-2,6-sialyltranferase 1
*STAB1*: Stabilin 1
*STK39*: Serine/threonine kinase 39
T2D: Type 2 diabetes
*TAOK3*: Thousand-and-one amino acid kinase 3
*TAPBP*: Tapasin binding-protein
*TAS2R40*: Bitter taste receptor 40
*TBCD*: Tubulin folding cofactor D
*TCF7L2*: Transcription factor 7 like 2
*TEX41*: Testis expressed 41 TFAP2E, Transcription factor AP-2 epsilon TMEM49 (VMP1)*,* Transmembrane protein 49 (Vacuole membrane protein 1)
*THADA*: THADA armadillo repeat containing
*TNNI3K*: TNNI3 interacting kinase
*TNNT3*: Troponin T3, fast skeletal type TPM2, Tropomyosin 2
*TUB*: TSD: Total sleep deprivation
*TUB*: Bipartite transcription factor
*UBASH3A*: Ubiquitin associated and SH3 domain containing A
*VEZT*: Vezatin, adherens junctions transmembrane protein
*VIPR2*: Vasoactive intestinal peptide receptor 2
VPA: Valproic acid
WC: Waist circumference
*WDR51A* (*POC1A*): WD repeat domain 51A (centriolar protein A)
*XYLT1*: Xylosyltransferase 1
*ZNF45*: Zinc finger protein 45
*ZNF608*: *Z*Inc finger protein 608
*ZNF643* (*ZFP69B*): Zinc finger protein 643 (*ZFP69B*)
